# Supplementary material for: Strengthening primary health care in Ethiopia: A scoping review of successes, challenges, and pathways towards universal health coverage using the WHO monitoring framework
Source: PLOS Glob Public Health. 2025 Apr 17;5(4):e0004470. doi: 10.1371/journal.pgph.0004470 (PMC12005562; doi:10.1371/journal.pgph.0004470)
Supplement: S1 Table — (DOCX) [file pgph.0004470.s001.docx]

S1 Table. Search strategies

| **Database** | **Search query** | **# of citations** |
| --- | --- | --- |
| PubMed | ("integrated health service*"[Title/Abstract] OR "multisectoral polic*"[Title/Abstract] OR "multi sectoral polic*"[All Fields] OR "multisectoral action*"[Title/Abstract] OR "multi sectoral action*"[Title/Abstract] OR "Primary health care"[Title/Abstract] OR "primary healthcare"[Title/Abstract] OR "primary care"[Title/Abstract] OR "community involvement"[Title/Abstract]) AND "health"[Title/Abstract] AND "Ethiopia*"[Title/Abstract] | 412 |
| Embase | (("integrated health service*" or "multisectoral polic*" or "multi sectoral polic*" or "multisectoral action*" or "multi sectoral action*" or "Primary health care" or "primary healthcare" or "primary care" or "community involvement") and Ethiopia*). ti,ab. | 552 |
| Scopus | TITLE-ABS-KEY ("integrated health service*" OR "multisectoral polic*" OR "multi sectoral polic*" OR "multisectoral action*" OR "multi sectoral action*" OR "Primary health care" OR "primary healthcare" OR "primary care" OR "community involvement”) AND TITLE-ABS-KEY ( "Ethiopia" ) AND ( LIMIT-TO ( DOCTYPE , "ar" ) OR LIMIT-TO ( DOCTYPE , "re" ) ) AND ( LIMIT-TO ( LANGUAGE , "English" ) ) | 730 |
| Web of Science | ((TS=('integrated health service*') OR TS=('multisectoral polic*') OR TS=('multi sectoral polic*') OR TS=('multisectoral action*') OR TS=('multi sectoral action*') OR TS=('Primary health care') OR TS=('primary healthcare') OR TS=('primary care') OR TS=('community involvement')) AND TS=('health') AND TS=('Ethiopia*')) and Article (Document Types) and English (Languages) | 1746 |
| CINAHL | ("integrated health service*" OR "multisectoral polic*" OR "multi sectoral polic*" OR "multisectoral action*" OR "multi sectoral action*" OR "Primary health care" OR "primary healthcare" OR "primary care" OR "community involvement") AND "health" AND "Ethiopia*" | 217 |
