# Supplementary material for: Strengthening primary health care in Ethiopia: A scoping review of successes, challenges, and pathways towards universal health coverage using the WHO monitoring framework
Source: PLOS Glob Public Health. 2025 Apr 17;5(4):e0004470. doi: 10.1371/journal.pgph.0004470 (PMC12005562; doi:10.1371/journal.pgph.0004470)
Supplement: S2 Table — (DOCX) [file pgph.0004470.s002.docx]

S2 Table. Characteristics and key findings of the included studies

| **Study ID** | **Study Design** | **Population/Participants** | **Sample Size** | **Outcomes** | **Population** | **Prevalence/Proportion** | **Key Findings** | **Conclusion** |
| --- | --- | --- | --- | --- | --- | --- | --- | --- |
| Alamneh et al. (2022)[1] | Cross-sectional surveys | Reproductive aged women | 4,772 | Incomplete utilisation of maternal continuum of care | Women in Ethiopia | Incomplete utilization = 94.83% | Rural residency, secondary education, higher education, religion, wealth index, barriers to healthcare access, mass media exposure, current working status | Public health interventions are crucial for maternal service utilisation and women's empowerment in hotspot areas. Significant hotspot areas were identified in the Somali, East Oromia, southeast part of the Southern Nations, Nationalities, People's Region, and Central Amhara regions of Ethiopia. |
| Karim et al. (2015)[2] | Cross-sectional surveys | Women from 129 communities | 3,932 (2008), 3,867 (2010) | Access to health facilities in L10K areas | Women in 115 districts of rural Ethiopia | Proportion within 30 min increased from 54% to 63%; >1 hour decreased from 22% to 9% | Significant improvement in access to health facilities in L10K areas over survey periods | Progress in enhancing geographical access to health services in rural Ethiopia, particularly within L10K program areas |
| Hierink et al. (2023)[3] | Cross-sectional surveys | Population in the Somali region | Not specified | Accessibility and availability of primary healthcare | The Somali region, Ethiopia | 65% lack access to health centers within a 1-hour walk, lengthy referral times, low health worker density  95.4% of health centers are understaffed in at least 6 out of 11 health worker categories; severe shortages in pharmacists and laboratory technicians  Only 29.6% of health posts meet staffing requirements, and over 50% have only one health extension worker. | Significant challenges in healthcare accessibility and worker scarcity in Somali region; recommendations for facility upgrades and outreach strategies | Improving primary healthcare accessibility and workforce availability is crucial for implementing the healthcare roadmap in Ethiopia's |
| Tadesse et al. (2019)[4] | Cross-sectional survey | Health centers and health posts in CORE Group Polio Project implementation districts | 860 PHCUs | Immunisation service availability and readiness | Pastoral and semi-pastoral regions of Ethiopia's implementation districts | Immunization service provided = 92%; Service readiness = 56.6% overall; Functional refrigerators in PHCUs = 65% | Uneven readiness observed among health centers and health posts; significant gaps in service delivery | Recommendations include ensuring all PHCUs have functional refrigerators, maintaining regular immunisation service availability, and providing supervisory support for cold chain management and service delivery |
| Drown et al. (2024)[5] | Implementation research | Communities benefiting from the Health Extension Program (HEP) | Not specified | Under-5 mortality reduction through community health program | Ethiopia, rural and pastoralist communities | PCV-10 coverage (three doses): 76%, with high acceptability (nearly 100%) | HEP leveraged for national evidence-based implementations (EBIs) (PCV-10, iCCM); improved care-seeking behaviours | HEP effectively integrates and scales evidence-based implementations (EBIs), addressing U5M; challenges remain in equity for pastoralist and rural populations |
| Fentie et al. (2023)[6] | Cross-sectional survey | Female youths aged 15-24 years in Ethiopia | 6,143 | Barriers to healthcare access | Female youths in Ethiopia | 61.3% (95% CI 60.1, 62.5) of Female youths had barriers to healthcare access due to the most frequently mentioned barriers – financial difficulty and distance from health facilities | Risk factors associated with healthcare access in youths include:  Age 15-19 (0.80, 95% CI 0.68-0.95)  No formal education (2.26, CI 1.61-3.18)  Primary education (2.21, CI 1.66-2.95)  marital status (1.43, 95% CI 1.21-1.70)  poor household wealth (1.63, 95% CI 1.31-2.05)  no media exposure (1.67, 95% CI 1.41-1.98)  rural residency (1.63, 95% CI 1.05-2.54)  low community media exposure (1.45, 95% CI 1.01-2.08) | Barriers to healthcare access disproportionately affect youth, particularly those with lower education, rural residency, and poor household wealth. Interventions should focus on economic improvement, media exposure, and educational access to reduce barriers. |
| Zimmerman et al. (2019)[7] | Longitudinal survey | Postpartum women in SNNPR, Ethiopia | 329 screened, 307 completed surveys at six months postpartum | Postpartum family planning (PPFP) counselling and uptake | Women in SNNPR, Ethiopia | Coverage of PPFP counselling (by six weeks) = 20% | PPFP is associated with contraceptive uptake (HR: 3.4, p < .01). Both antenatal and postnatal care counselling are effective in increasing PPFP (HR: 2.5, p = .01) | Integrating PPFP counselling into postnatal care enhances postpartum contraceptive uptake, but gaps persist in coverage, particularly during delivery and postnatal periods. |
| Tiruneh et al. (2021)[8] | Cross-sectional study | Women with children 0–11 months | 51 IDIs were conducted with WDAs, HEWs, health centre directors, health centre staff L10K 2020 QI specialists | Changes in maternal and newborn healthcare indicators | 39 intervention communities, 148 comparison communities | Not provided | Skilled delivery care: 7.9% increase (1.8–13.9%) Postnatal care within 48 hours: 15.3% increase (7.4–23.2%) | Community participation improves the use of high-impact maternal and newborn health services and supports participatory strategies for community-responsive health systems. |
| Astale et al. (2015)[9] | Cross-sectional study | Children aged 0-59 months with acute respiratory infection | 11,030 | Help-seeking behaviour for acute respiratory infection | Mothers aged 15-49 years in Ethiopia | 7% reported symptoms | Rural residence: OR 1.6 (95% CI: 1.2-2.0) Urban: 46.4%, Rural: 25.2% sought treatment | Rural residents are associated with a higher risk of acute respiratory infection and lower help-seeking behaviour. |
| Ketema et al. (2023)[10] | Cross-sectional | Women attending rural healthcare facilities in South Central Ethiopia | 420 | Intention to undergo clinical breast examination (CBE) | Women aged >18 years in rural Ethiopia | 90% never had a clinical breast examination (CBE) | Positive attitudes and subjective norms correlate with higher rates of clinical breast examination (CBE). Conversely, lower wealth and spouses' education (unable to read/write) are linked to lower CBE utilisation. | A high intention score suggests readiness for clinical breast examination (CBE); interventions should focus on improving attitudes and norms and addressing educational disparities among spouses. |
| Abate et al. (2023)[11] | Qualitative study | Adult cancer patients, primary caregivers, healthcare providers, volunteers, nationwide advocates | 25 participants for in-depth interviews, including patients and primary caregivers | Barriers to continuity of palliative care from facility to household | Cancer patients in Addis Ababa, Ethiopia | Not applicable | Lack of access to primary and specialised palliative care.  There is no established structure for implementing palliative care continuity.  Key barriers: opioid scarcity, turnover of healthcare workers, diagnostic materials shortage, medication costs, lack of government support, cultural barriers, patient preference for conventional medicine, failure of health extension workers, and healthcare professional workload. | The palliative care continuum in Ethiopia faces challenges related to availability, accessibility, acceptability, utilisation, and effectiveness. |
| Bekele et al. (2023)[12] | Mixed methods | Primary healthcare clinicians, healthcare administrators | 10 primary care clinicians + 124 health workers | Barriers and enablers to implementing Ethiopian Primary Healthcare Clinical Guidelines (EPHCG) for NCDs-MHCs | Health facilities in southern Ethiopia | Not applicable | Key barriers include resource constraints (e.g., lack of reagents and medications), care pathway bottlenecks, and socio-economic challenges. | Practical implementation of EPHCG for NCDs-MHCs faces significant challenges across various domains. |
| Endris et al. (2023)[13] | Qualitative study | Key stakeholders and service providers in four districts of Ethiopia | 42 key informant interviews | Barriers and facilitators to implementing nutrition services for children | Health facilities | Not applicable | Barriers include a lack of anthropometric equipment, a high caseload, poor staff motivation, non-functional health posts, false reporting, and a lack of leadership support. | Effective implementation requires improving staff motivation, leadership engagement, and system strengthening for monitoring and supervision. |
| Enyew et al. (2017)[14] | Quantitative descriptive | Women, midwives, Health Extension Workers (HEWs) | 304 women interviewed; opinions from all midwives and HEWs in three health centers and 15 health posts | Contributions of the Health Extension Programme (HEP) to maternal healthcare service utilisation | Mecha District, Amhara Region, Ethiopia | Not applicable | Ineffective continuum of care for maternal healthcare services; poor quality of documentation; inadequate advocacy and dissemination of maternal healthcare services | The contributions of health extension programmes (HEP) to maternal healthcare service utilisation are erratic in terms of the continuum of care. |
| Aregay et al. (2023)[15] | Qualitative study | Health professionals, community members, health planners and practitioners | Not specified | Palliative care in rural and regional health care settings: enabling factors and implementation challenges | Ethiopia, rural and regional healthcare settings | Not applicable | Palliative care is not well integrated into Ethiopia's health care system. | Enablers include better integration into national health plans, inclusion in academic courses, and use of mobile technology. |
| Aregay et al. (2023)[16] | Qualitative study | Policymakers, pharmacists, medical doctors, health officers, nurses | 42 participants | Perceived policy-related barriers to palliative care implementation in rural and regional healthcare settings | Resident of Rural region of Ethiopia | Not applicable | Key barriers to integration include a lack of government priority for palliative care, low awareness among health professionals about national guidelines, and inadequate integration into healthcare systems and budgets. Palliative care services are mainly limited to HIV patients with external support, which is unsustainable. | Policy priority and integrating palliative care into all healthcare levels, especially primary healthcare units and the health extension programme, are crucial for improving access and sustainability of palliative care services in Ethiopia's rural and regional settings. |
| Gesesew et al. (2020)[17] | Qualitative study | HIV care providers, researchers, HIV programme managers | 18 experts participated | Policy and practice suggestions to improve performance on the UNAIDS 90-90-90 targets in Southwest Ethiopia | Experts from Southwest Ethiopia | Not applicable | Priority solutions include filling gaps in legislation, HIV self-testing, the teach-test-link-trace strategy, house-to-house HIV testing, community ART groups, providing ART in private clinics, and providing ART at health posts. | Mandatory partner notification of HIV status, HIV self-testing, and involvement of peer educators are recommended to enhance the effectiveness of HIV care programmes in addressing adverse outcomes. |
| Shimels et al. (2021)[18] | Mixed-methods study | Health facilities, key informants (healthcare providers) | Not specified | Trend of health service utilisation and challenges during the COVID-19 pandemic at primary units in Addis Ababa, Ethiopia | Five health centers located in Addis Ababa, Ethiopia | Not applicable | There was a significant drop in inpatient flow and services such as PICT, VCT, FP, and OPD units during the COVID-19 pandemic. Challenges included fear of infection and stigma, poor infrastructure, human resource challenges, and supply shortages. | The COVID-19 pandemic severely impacted service delivery at primary healthcare units in Addis Ababa, highlighting the need for improved infrastructure, adequate PPE supply, and comprehensive support to ensure uninterrupted services. |
| Argaw et al. (2020)[19] | Mixed-methods study | District health systems, stakeholders involved in twinning partnerships | Eight districts | Accelerating district health system performance towards achieving Universal Health Coverage (UHC) via twinning partnerships | Amhara, Oromia, SNNP, and Tigray regions, Ethiopia | Not applicable | Significant improvement in District Health System Performance (DHSP) scores from baseline (50.97) to midterm (60.3) and end-line (72.07). A strong positive relationship exists between DHSP scores across measurement points (r > 0.978**). Qualitative findings highlighted inputs for successful partnerships: shared vision, resource mobilisation, skilled leadership, clear roles, detailed action plans, and effective communication. | Twinning partnerships effectively enhance district health system performance to meet transformation criteria for UHC. Scaling up twinning partnership strategies is recommended to accelerate progress in Ethiopian districts. |
| Badacho et. al (2023)[20] | Qualitative study | Healthcare providers and managers from primary health facilities | 15 key informant interviews | Facilitators and barriers to integration of hypertension and diabetes with HIV care at primary healthcare | Wolaita zone, South Ethiopia | Not applicable | Facilitators: Perceived benefits to patients and providers, availability of guidelines, supportive policy, leadership commitment. Barriers: Increased cost, less attention to NCD care, lack of trained professionals, inadequate equipment, budget allocation, and poor data culture. | Addressing contextual barriers through innovative implementation  is vital to address barriers and meet the DM and hypertension care needs of people living with HIV (PLWH). Integrating hypertension and diabetes care with HIV care in primary healthcare is essential. |
| Donovan et al. (2023)[21] | Mixed methods study | Health workers and facilities in Damot Gale district, Wolaita Zone, Ethiopia | One hospital, one health centre, five health posts | Evaluation of an intervention integrating four NTDs (trachoma, lymphatic filariasis, schistosomiasis, podoconiosis) into primary healthcare | Damot Gale district, Wolaita Zone, SNNPR, Ethiopia | Not applicable | Improved detection, management, and reporting of NTDs; high level of acceptance from health workers; intervention demonstrated cost-effectiveness | Integrating NTD services into primary healthcare is feasible, acceptable, and cost-effective, requiring further investment and scaling up. |
| Mayston, R., et al. (2016)[22] | Participatory planning approach | Healthcare administrators, providers, caregivers, service users, community leaders | Four community consultation meetings, seven focus group discussions, 11 in-depth interviews | Is that feasible and acceptable to transition from hospital-based psychiatric care to a primary care-based, task-sharing model for people with severe mental disorders (SMD)? | Rural Ethiopian community | Not applicable | Increased access due to proximity, reduced cost, reduced transportation stress, concerns about staff competence, need for adequate training, support, and consistent medication supply, significant support for service change and community resource mobilisation. | Significant support for transitioning to a primary care-based model with the need for training, support, and consistent medication supply is identified as a key component for success. |
| Tadesse, E., et al. (2016)[23] | Mixed-methods study | Caregivers of children admitted to the OTP, Health Extension Workers (HEWs) | 1048 caregivers, 175 HEWs from 94 health posts | Challenges in implementing critical steps in Community-based Outpatient Therapeutic Program (C-OTP) and caregivers' perceptions of service provision in southern Ethiopia | Rural southern Ethiopia | Children given recommended RUTF = 46.6%  Children given antibiotics = 19.3%  Children given recommended RUTF during follow-up = 49.9%  Children with adequate/uninterrupted RUTF = 34.9%  HEWs reporting lack of RUTF = 32.9%  HEWs without antibiotics available = 62.9%  Children exited from the RUTF program = 39.7% | Key challenges in implementing integrated Community-based Outpatient Therapeutic Programs (C-OTPs) include inadequate provision and misuse of Ready-to-Use Therapeutic Food (RUTF), inconsistent availability of antibiotics, and inadequate home visits by Health Extension Workers (HEWs). | Addressing these challenges requires ensuring reliable RUTF supply chains, implementing interventions to address food insecurity, providing regular training for healthcare providers, improving antibiotic availability, and establishing clear protocols for patient transfers. A collaborative policy approach involving local governments and various stakeholders is essential for overcoming these barriers and enhancing the effectiveness of C-OTPs in managing severe acute malnutrition. |
| Datiko, D. et al. (2019)[24] | Mixed-methods study | HEWs, HEW supervisors, maternal health program managers, community members | 32 HEWs, eight supervisors, program managers, 8 FGDs with community members, quantitative data analysis | Effect of intervention on maternal health service utilisation and stakeholders' perceptions of community participation efforts | Rural Southern Ethiopia | HDA leaders' attendance increased by 30.6%,  PWF attendance increased by 36%  Antenatal care utilisation increased from 73.4% to 77.6%  Skilled delivery from 76.7% to 83.3% | Challenges in providing training and supportive supervision for HEWs included lack of incentives, absenteeism, and limited support from kebele administrators. | Focused training, guidance, and regular supervision for HEWs can improve community participation and maternal health service utilisation in rural communities. |
| Curry, L. A., et al. (2019)[25] | Qualitative study | Community members in Amhara and Oromia, Ethiopia | 14 focus groups per 7 primary health care units, 140 participants total | Community perspectives on roles and responsibilities in strengthening primary health care | Rural Ethiopia | Not applicable | Community roles: promoting health behaviours, influencing social norms, contributing resources; Government roles: overseeing health centers, providing resources and support for community health workers | Community participation is essential in strengthening primary health care, with defined roles for communities and governments; partnerships require insights from community perspectives for effective and sustainable health initiatives. |
| Tolera, H., et al. (2020)[26] | Bivariate and multivariable logistic regression analyses | Mothers in Gida Ayana Woreda, rural western Ethiopia | 454 | Risk factors for non-utilization of decentralised health facilities for postnatal care | Rural western Ethiopia | 55.7% did not utilise postnatal care, 10% used care as per WHO guidelines | Risk factors for non-utilization: older age (35+), no antenatal care, no postnatal complications, lack of knowledge about postnatal complications, services, or recommended visits, and lack of home visits from health extension workers by day three post-delivery. | Interventions should target identified risk factors to enhance postnatal care utilisation and reduce maternal and newborn deaths, including strengthening services in remote kebeles and expanding health worker counselling. |
| Tekola, B., et al. (2023)[27] | Qualitative study | Primary care attendees with depression and community members (males, females, priests) in rural Ethiopia | 49 (28 interviews, 21 FGDs) | Exploration of how depression is conceptualised and communicated among community members and primary care attendees with depression | Rural Ethiopia | High prevalence in primary care settings, often unrecognised | Community members did not identify depression as a mental illness, viewing depressive symptoms as normal reactions to life stressors. Medical intervention was considered only when symptoms severely affected the mental state. Attendees with depression viewed their condition as an illness, with spiritual and traditional healing prominent in explanations. | Understanding local conceptualisations of depression is crucial for improving detection and negotiating acceptable interventions for affected individuals. |
| Shiferaw et al., 2019[28] | Mixed-method study | Data from Ethiopia NCD STEPwise approach to surveillance (STEPS) survey; national policies and strategies | Not specified | Examination of policy and strategy gaps in reducing modifiable NCD behavioural risk factors | Ethiopia | Higher risk of NCDs, including | Substantial progress in developing the health sector NCD strategic action plan and setting national targets; ongoing activities to reduce tobacco use. Identified gaps in policy and strategy, law enforcement, social mobilisation, and awareness creation for reducing behavioural risk factors | There is an urgent need to develop and implement targeted strategies for each behavioural risk factor and design functional, multisectoral coordination. Establishing sustainable financial mechanisms is essential to support NCD prevention and control. |
| Awol et al., 2023[29] | Multilevel logistic regression analysis | Women who had live births in Ethiopia | 5,753 | Determination of spatial patterns and factors affecting institutional delivery | Ethiopia | Institutional delivery: 26%; Maternal mortality: 412 deaths/100,000 live births | Individual factors (education level, media exposure, antenatal visits, wealth, birth interval) and community factors (high antenatal visit proportion, region) are significantly associated with institutional delivery. | Community education and health worker involvement are crucial for promoting institutional delivery, focusing on antenatal care and regional disparities. |
| Atnafu, A., et al. (2020)[30] | Cross-sectional study | Mothers who gave birth five years before the study | 565 | Factors associated with completion of maternal healthcare services | Northwest Ethiopia | Completion of maternal healthcare services: 21.60% (95% CI: 18.20, 24.90) | Literacy, transport access, shorter travel time, service satisfaction, and recent health education were positively associated with service completion | Interventions should increase women's awareness, improve service availability, and enhance service delivery by considering women's preferences and needs. |
| Adane et al., (2020)[31] | Cross-sectional study | Women who gave birth two years before the survey in Ethiopia | 4489 | Factors associated with postnatal care (PNC) utilisation | Ethiopia | Four or more ANC visits = 33.30%  No ANC visit = 35.54%  Caesarean section birth = 97.34%  Low autonomy reported = 11.47%  Factors  Husband’s secondary education [AOR = 0.17, 95% CI = (0.04, 0.68)]  Four or more antenatal care visits [AOR = 10.77, 95% CI = (2.65, 43.70)]  Middle wealth quintile [AOR = 3.10, 95% CI = (1.12, 8.58)] | The husband's secondary education, ≥ four antenatal care visits, and middle wealth quintile were individual factors. Community-level education and health service utilisation were community-level predictors. | Enhancing antenatal care quality, promoting women's education, involving husbands in PNC, and improving community health service utilisation can boost PNC utilisation. |
| Memirie et al., (2017)[32] | Facility-based study | Children (0-59 months) with pneumonia and diarrhoea in Ethiopia | 345 pneumonia, 341 diarrhoea | Household expenditures on pneumonia and diarrhoea treatment in Ethiopia | Ethiopia | 7% and 6% impoverished | Mean outpatient medical costs: US$8 (pneumonia), US$6 (diarrhoea); mean inpatient costs: US$64 (severe pneumonia), US$79 (severe diarrhoea). Wealthier, urban households had higher OOP payments. Poorer, rural families are more likely to be impoverished. | High costs for treating childhood pneumonia and diarrhoea in Ethiopia lead to catastrophic health expenditures and impoverishment. Health financing strategies need to be reevaluated to alleviate financial burdens on households. |
| Girmaw et al., (2023)[33] | Cross-sectional | Government healthcare professionals in North Wollo Zone, Amhara Region, Ethiopia | 636 | Willingness to Pay (WTP) for Social Health Insurance (SHI) among healthcare professionals | Ethiopia, North Wollo Zone | 61.7% willing to join SHI | Factors influencing WTP included having children under five, gender (female), and increasing monthly salary. Qualitative factors included premium amount, benefits, and service quality. | The majority of healthcare professionals in North Wollo Zone, Ethiopia, are willing to pay for SHI, indicating the feasibility of healthcare financing reform for SHI implementation. To ensure successful implementation, efforts should focus on addressing factors influencing WTP. |
| Beshah et al., (2023)[34] | Program cost analysis | Adult primary care users in Ethiopia | Not specified | Costs of WHO-HEARTS program for hypertension and CVD prevention | Ethiopia | Not applicable | The estimated costs are USD 5.3 for hypertension control and USD 19.3 for integrated CVD risk management—medication costs: USD 9.0 for hypertension, USD 15.4 for diabetes, and USD 15.3 for high cholesterol. Medications were significant cost drivers. | Results inform planning and budgeting for scaling up WHO-HEARTS to prevent CVD in Ethiopia. |
| Assefa et al., (2020)[35] | Document review | Ethiopian health system | Not applicable | Role of primary health care towards universal health coverage (UHC) | Ethiopia | Not applicable | Ethiopia's primary health care integrates policies, disease control, community empowerment, and multisectoral action, enhancing service coverage. Challenges include access disparities, noncommunicable disease adaptation, care quality, and high costs. | Ethiopia's primary health care strategy has advanced health service coverage and outcomes but faces critical challenges that necessitate enhanced funding and targeted approaches to achieve sustainable development goals. |
| Assebe, L. F., et al. (2021)[36] | Facility-based cross-sectional | Rural households in Jimma Zone, Oromia Region | 221 | Financial risks of malaria service-seeking | Rural households in Jimma Zone, Oromia Region | 12% incurred catastrophic health expenditure (CHE); 40% of the poorest quintile experienced CHE | The average cost of malaria services was US$4.40; 52% of costs were indirect. Poorest households faced significant financial risks. | Malaria care seeking imposes substantial financial burdens, particularly for the poorest. Policy interventions should mitigate economic impacts, focusing on vulnerable groups. |
| Arage et al. (2022)[37] | Cross-sectional | Health professionals in primary healthcare facilities, Addis Ababa, Ethiopia | 459 | Level of organisational commitment and associated factors | Addis Ababa, Ethiopia | Organisational commitment mean score was 48.4% | Risk factors for low organisational commitment Age (>30 years), job satisfaction, and good transformational leadership were significant predictors. | Organisational commitment in primary healthcare facilities in Addis Ababa needs enhancement. Age, job satisfaction, and leadership are critical factors to address. |
| Argaw et al. (2021)[38] | Cross-sectional study | Health workers in primary healthcare entities, Amhara, Oromia, Tigray, and SNNP regions, Ethiopia | 454 | Comparison of maternal and child health service performances following LMG intervention | Primary healthcare entities in Ethiopia | Contraceptive acceptance rate = 3.54%  Antenatal care =3.51%  Skilled birth attendance = 2.64%  Postnatal care = 3.0% Full immunization = 1.07%  Growth monitoring services = 3.34% | LMG intervention is associated with higher performance in maternal and child health indicators compared to the non-exposed group. Significant improvements in management systems and work climate were observed. | LMG interventions enhance maternal and child health service performances and strengthen health systems in primary care settings in Ethiopia. |
| Bayou et al. (2020)[39] | Mixed methods study | Healthcare workers in 12 health centers, sub-city health offices, and town health offices in Ethiopia | Not specified | Impact of LMG training on leadership competencies and primary healthcare service delivery | Health facilities in Ethiopia | Institutional delivery: 40% to 80% in Sodo town; ANC follow-up retention rate: 40% to 78% in Addis Ababa Woreda10 HC | LMG training improved the workplace atmosphere, provider motivation, teamwork, and resource management. It also had a positive impact on service delivery. | Scaling LMG training can catalyse health sector transformation in Ethiopia by enhancing managerial and provider competencies. |
| Berhan et al. (2022)[40] | Critical interpretive analysis | Not applicable | Not specified | Status of universal access to Sexual and Reproductive Health and Rights (SRHR) and progress towards Universal Health Coverage (UHC) in Ethiopia | Ethiopian population | Not applicable | SRHR is being integrated into the UHC benefit package with significant progress. However, other SRHR services, such as abortion care, FGM treatment, CSE, and sexual health services, are not fully integrated. Capital-intensive SRH services are also excluded from the UHC benefit package. | Domestic financing is crucial for expanding SRHR services under UHC to avoid financial hardship. Multi-sectoral efforts are needed to strengthen health systems and achieve the SDGs. |
| Chantler et al. (2018)[41] | Formative evaluation | Caregivers, health workers, community members/leaders | 18 participant observations, 46 semi-structured interviews, and six focus groups | Community engagement strategy ('The Fifth Child Project') in northwest Ethiopia to improve immunisation uptake. | North-west Ethiopia | Not specified | Integration of CE strategy with health extension program; practical defaulter-tracing system established. Colour-coded health calendars facilitated personalised health discussions. Sanction exercise was observed as a deterrent to vaccine default. | The CE strategy facilitated personalized health discussions and improved defaulter tracing in immunisation services. It emphasises the need for transparency and community engagement in health discussions. |
| Desta et al. (2020)[42] | Comparative cross-sectional | District health offices in Ethiopia | LMG: 94, non-LMG: 190 | Comparison of district-level capacity and performance between LMG and non-LMG districts in Ethiopia. | Ethiopia | LMG districts showed statistically better average performances compared to non-LMG districts (61.8 ± 121.45 vs. 56.89 ± 110.39 SD, p < 0.001). | LMG districts benefited from leadership, management, and governance interventions supported by USAID. | Leadership development programs at the district level improve capacity, management practices, and the quality of care in Ethiopian health systems. |
| Fetene et al. (2020)[43] | Qualitative study | Managers at all levels of Ethiopia's primary healthcare system | 41 key stakeholders | Characterisation of how managers experience accountability in Ethiopia's primary healthcare system | Ethiopia | Not applicable | Development of shared understanding of system-wide accountability, streamlining of managerial reporting lines, strengthening of medico-legal knowledge and systems, and bottom-up accountability mechanisms where themes emerge to improve accountability in primary healthcare | These themes are relevant for policymakers, practitioners, development partners, and researchers aiming to enhance accountability frameworks and practices in public health systems. |
| Hailemichael et al. (2021)[44] | Cross-sectional study | Referral service beneficiaries or adult caregivers | 1,139 | Assessment of referral barriers at primary healthcare entities in Ethiopia | Ethiopia | 95% of formally referred patients reported no pre-referral communication between referring and receiving facilities | Lack of provider communication skills, absence of governing documents, human resource shortage, lack of essential ambulance equipment, and inadequate infrastructure are some of the challenges. | Several referral service barriers were observed in four regional states of Ethiopia. Both individual and health system factors influenced the referral service barriers. |
| Heyi et al. (2022)[45] | Facility-based comparative | Health workers in project-supported and non-supported PHCs | 364 | Excellence in PHCs following performance management and organisational culture innovations | Oromia and SNNP regions, Ethiopia | The mean baseline primary healthcare unit excellence score was 63.2% and 50.5% for project-supported and non-supported health facilities, respectively.  The end-line excellence scores increased to 93.3% for project-supported and 79.1% for non-supported facilities. | Project-supported health workers showed significantly higher perceived organisational culture scores (p = 0.001). Excellence scores increased from baseline to end line: 63.2% to 93.3% in project-supported vs. 50.5% to 79.1% in non-supported PHCs. | Implementing performance management innovations correlates with enhanced organisational culture and excellence in PHCs. Scaling up these interventions is crucial for achieving Universal Health Coverage (UHC) through high-performing primary healthcare facilities. |
| Liu et al. (2022)[46] | Mixed-method study | Health centers, districts, and zones in Ethiopia | 315 districts, 1617 health centers | Evaluation of management capacity and healthcare performance in Ethiopia's Primary Healthcare Transformation Initiative (PTI) | 19 zones in Ethiopia | Zonal adherence to management standards improved by 37% (P<.001), district by 18% (P<.001), and health centers by 18% (P<.001). Performance summary score improved by 14% (P<.001). | Improvement in zonal-level management practices correlated with significant enhancements in district-level management and healthcare performance. District management mediated the relationship between zonal and health centre levels. | Enhancing managerial practices at the zonal level can effectively boost primary healthcare system performance at scale in low-income settings like Ethiopia. Investment in zonal-level management is crucial for sustained healthcare improvements. |
| Liu et al. (2020)[47] | Longitudinal study | District health offices and health centers in Ethiopia | 36 districts, 229 health centers | Evaluation of management capacity and healthcare performance in Ethiopia's Primary Healthcare Transformation Initiative (PTI) | Rural districts in Ethiopia | Adherence to management standards at district and health centre levels improved over the intervention period. No significant change was observed in the KPI summary score. | Management capacity at district and health centre levels improved significantly with intensive mentorship and education. Primary healthcare performance indicators did not show a corresponding improvement. | District health offices are pivotal for initiating primary healthcare reforms. Improvements in management capacity can be achieved through intensive mentorship and education. These approaches can catalyse broader healthcare system reforms despite the limited impact on performance indicators during the study period. |
| Abajebel et al. (2011)[48] | Cross-sectional | Heads/units/departments of the District Health Office, Health Center, and Health Post | 362-unit heads composed of 10 from the districts, 13 From Health Centers and one unit from each Health Post participated in this study | Assessment of health information system utilisation at district level | Jimma Zone, Ethiopia | Health information system utilisation was only 32.9% across | Documentation practices and performance monitoring significantly influenced utilisation. | The study highlighted poor implementation and utilisation of health information systems at district and primary health unit levels. |
| Asemahagn, M. A. (2017)[49] | Cross-sectional | Health centre and department/unit heads in East Gojjam Zone, Northwest Ethiopia | 250 | Determinants of routine health information utilisation | East Gojjam Zone, Ethiopia | Only 38.4% of participants routinely used health information for multiple purposes beyond reporting duties. | Factors like residence, data management knowledge, workload, and access to HMIS resources influenced utilisation. | The study highlights the low utilisation of health information at health centers. Addressing personal (skills, knowledge) and organisational (access, training, supervision) factors is crucial to improving information use. |
| Ayele, W., et al. (2021)[50] | Cross-sectional study | Public health centers in Addis Ababa, Ethiopia | Nine health centers (randomly selected) from three sub-cities | Patterns of essential health service utilisation, data quality, and performance review practice before and during COVID-19 | Addis Ababa, Ethiopia | Pneumonia service utilisation was reduced by 70%, Upper respiratory diseases by 65%, Provider-Initiated Counselling and Testing (PICT) by 54%, Outpatient services by 42%, and data quality and performance review practices by 39% during COVID-19. ANC1, ANC4, Penta1, and Penta4 services showed similar trends before and during COVID-19. | Significant reduction in service utilisation (emergency, outpatient, VCT cases), routine data accuracy checks, and RHIS performance review practices during COVID-19. | Essential health services and routine health information management were significantly disrupted during the COVID-19 pandemic, indicating the need for strategies to maintain health service delivery during crises. |
| Bogale, T. N., et al. (2023)[51] | Qualitative study | Health extension workers (HEWs), HEW supervisors, health information technicians, and managers | 54 in-depth interviews | Barriers, facilitators, and motivators of electronic community health information system (eCHIS) use | Amhara, Harari, Oromia, Sidama, Southwest Ethiopia, and Southern Nations Nationalities and People's regions, Ethiopia | Not applicable | Barriers: Lack of infrastructure and resources, poor quality of training, follow-up, and supervision, parallel manual and electronic recording, and HEWs' workload.  Facilitators: Data quality, retrievability, and traceability; tablet portability; supervisor encouragement; positive community image. | The study highlights various barriers and facilitators affecting eCHIS use among health workers. An integrated approach addressing these barriers and reinforcing facilitators is essential for effective eCHIS implementation. |
| Bramo, S. S., et al. (2023)[52] | Ethnographic study | Primary Level Health Care (PLHC) facilities | Not specified | Challenges in implementing ICT-Based Health Information system (ICT-BHIS) | Wolaita Zone, South Ethiopia | Not applicable | Key challenges include infrastructure issues, financial costs, technical constraints, human capital, stakeholder engagement, and organisational commitment. | A unified strategy addressing multiple challenges is needed for successful ICT-BHIS implementation in PLHC facilities. |
| Hailemariam et al. (2023)[53] | Qualitative study | Rural Wogera district, northwest Ethiopia | Not specified | Enablers and barriers in electronic community health information system (eCHIS)implementation | Not applicable | Not applicable | Implementers valued eCHIS but faced challenges: heavy workload, limited infrastructure, turnover, competing projects, lack of ownership and resources, digital literacy, and age barriers. Recommend mentoring, community engagement, and structured planning. | It emphasizes government commitment, resource allocation, institutionalization, capacity building, and continuous monitoring for successful and sustainable eCHIS implementation. |
| Kassa et al, (2022)[54] | Comparative study | 4 Health Centers in Ethiopia | 800 clients | Effectiveness of mHealth application to improve maternal and newborn health services | Maternal and newborn women | After intervention: ANC 4+ visits increase from 13.8% to 64%  Timely initiation of ANC increased from 44.5% to 77.3%  Institutional delivery from 35.0% to 71.2%  Penta-3 vaccination coverage increased from 61.5% to 70.4%. | Significant increase in antenatal care visits (mean 2.21 to 3.43) | mHealth intervention effectively enhances maternal and newborn health service utilisation, showing potential for scaling up and broader health interventions. |
| Kebede et al. (2020)[55] | Cross-sectional study | Health professionals/Health Information Technicians | 316 | Evaluation of HMIS data quality (timeliness, completeness, accuracy) | Primary health care units, East Wollega zone, Ethiopia | Timeliness of report: 70%  Registration completeness: 78.2% Report completeness: 86%  Data accuracy: 48% | HMIS data quality below national standards  Reasons: poor management support, lack of accountability, inadequate supervision | The health information management system is poorly coordinated at the primary health units. |
| Tadesse et al. (2021)[56] | Case Study | Ethiopian health system | Not specified | Efforts to synergise universal health coverage, health security, and health promotion | Ethiopia | Not applicable | Fragmentation of the health system: inequities, low health workforce, limited implementation capacity  Challenges: donor-driven vertical programs, diverse health financing modalities, inadequate multisectoral collaboration | Ethiopia has implemented various strategies to address fragmentation and enhance synergies among global health agendas, contributing to improved health indicators and sustained health system functionality. |
| Bisrat et al. (2019)[57] | Cross-sectional study | Healthcare providers involved in immunisation services in pastoral and semi-pastoral regions | 1,283 | Readiness to provide immunisation services | Pastoral and semi-pastoral regions in Ethiopia | Readiness to provide immunisation at the PHC level:  A higher level of readiness (61.1%) was observed among those who had a diploma (58.8%), nurses (62,4%), and those with more than five years of service (65.5%) | Significant factors influencing readiness: education level, job role, satisfaction with supportive supervision  Nurses and those satisfied with supervision are more ready than midwives and less satisfied counterparts  Gender and work experience also impact readiness among health post-staff | Improving in-service training and supportive supervision is critical to enhancing healthcare workers' readiness to deliver immunisation services in Ethiopia's pastoral and semi-pastoral regions, mainly targeting factors like education, job satisfaction, gender, and experience levels. |
| Tekle et al. (2022)[58] | Mixed methods study | Health Extension Workers (HEWs) in Ethiopia | Not specified | Attrition rate, intention to leave | HEWs in Ethiopia | Attrition: 21.1% (95% CI 17.5-25.3%)  Intention to leave: 39.5% (95% CI 32.5-47%) | Lack of COC, deployment after 2008, and having a diploma/degree were associated with higher HEP attrition rates. A lower attrition rate is linked with level four certification, having children, and an urban setting. | Strategies to enhance job satisfaction and address factors contributing to attrition are crucial to retaining HEWs and sustaining primary healthcare delivery in Ethiopia. |
| Tsigebrhan et al. (2021)[59] | Cross-sectional study | People with epilepsy and primary health care (PHC) workers in rural Ethiopia | 237 | Sensitivity, specificity, PPV, NPV of PHC worker diagnosis against standardised reference diagnosis | People with epilepsy in rural Ethiopia | Standardised reference diagnosis: 13.9%  PHC workers' diagnosis: 6.3% | Sensitivity and specificity of PHC workers' diagnosis: 21.1% and 96.1%, respectively  Combining SRQ-20 score ≥ 9 with PHC diagnosis of depression increased sensitivity to 78.9% (95% CI 73.4-84.4%) and specificity to 59.7% (95% CI 53.2-66.2%)  Older age is significantly associated with misdiagnosis (adjusted OR, 95% CI = 1.06, 1.02-1.11) | Routine detection of comorbid mental disorders in people with epilepsy by PHC workers is very low. Combining clinical judgment with a screening scale may improve detection rates, but further evaluation is needed to optimise this approach. |
| Badacho, A. S. et al., (2023)[60] | National sustainability assessment | People living with HIV at primary healthcare facilities in South Ethiopia | Not specified | Factors promoting sustainability: perceived benefit, adaptability, alignment with organisational goals | People living with HIV at primary healthcare facilities in South Ethiopia | Overall mean sustainability: 43.74 (95% CI: 42.15–45.33) | Integrating hypertension and diabetes with HIV care sustainably requires staff involvement and training, behaviour change communication, management and clinical leadership engagement, and addressing infrastructure limitations.  Challenges: lack of progress monitoring system, staff behaviour, inadequate training, senior leadership support, infrastructure limitations | Addressing organisational, training, and infrastructure challenges to sustainably integrate hypertension and diabetes care with HIV care at primary healthcare facilities in South Ethiopia is crucial. |
| Berhanu, A., et al. (2020)[61] | Cross-sectional | Sick under-five children with common childhood illnesses | 633 | Utilisation health post | Sick under-five children with common childhood illnesses in Southern Ethiopia | Service utilisation for health posts is 10.6% | Factors associated with health post utilisation include income (AOR = 2.99, 95% CI: 1.37-6.53), previous service utilisation (AOR = 6.66, 95% CI: 1.81-24.04), awareness of service availability (AOR = 4.74, 95% CI: 1.39-12.10), ownership of health insurance (AOR = 2.63, 95% CI: 1.45-4.76), distance to health post (AOR = 5.23, 95% CI: 1.69-10.19), type of illness (AOR = 2.97, 95% CI: 1.41-6.25) | Low utilisation rates of integrated community case management at health posts. Factors such as income, previous service utilisation, awareness of service availability, health insurance ownership, distance to health post, and type of illness significantly influence utilisation. |
| Bradley, H., et al. (2008)[62] | Cross-sectional study | VCT client records from Ethiopian reproductive health clinics | 30,257 | Integration modality: co-locating vs. same rooms vs. joint counselling  Client composition: typical vs. atypical family planning clients  Client-initiated HIV testing  Client HIV status | VCT clients at Ethiopian reproductive health clinics | Client-initiated HIV testing: 78.2% (young, single men), 80.6% (older, married women) | Integration at room- and counsellor levels increases the likelihood of clients initiating HIV testing (OR = 1.9-7.2).  Facilities integrating HIV and family planning services in the same rooms are 2-13 times more likely to serve atypical family planning client types than older, ever-married women. | Integration of HIV and family planning services, particularly at room- and counsellor levels, attracts a diverse clientele, including high-risk groups, suggesting effective strategies for expanding VCT service delivery in resource-poor settings. |
| Gebremedhin, L. T., et al. (2021)[63] | Qualitative study | Health professionals in Ethiopia | Not specified | Integration of Mental Health and Substance Abuse (MH/SA) services into primary health care  Implementation of Ethiopian Primary Health Care Clinical Guidelines  Training of Health Extension Workers (HEWs) and health center staff in MH/SA care | Health facilities in Ethiopia | Not specified | Ethiopia integrated MH/SA services at all government levels, emphasising primary health care and training over 5,000 urban HEWs in MH/SA care.  Lessons learned include stakeholder buy-in, gradual integration to avoid "task dumping," and the importance of supervision and mentorship. | Lessons from Ethiopia's experience integrating MH/SA services are transferable to other countries. They emphasise the need for consistent leadership support, careful integration strategies, and ongoing training for sustainable care delivery. |
| Sitrin, D., et al. (2020)[64] | Quasi-experimental study | Pregnant and postpartum women | 772 pregnant women enrolled | Adoption of postpartum family planning (PPFP) | Pregnant and postpartum women in Ethiopia | PPFP adoption among women who delivered at home (Intervention: 35.2%, 95% CI: 28.8-42.4%; Comparison: 27.8%, 95% CI: 22.2-34.4%) | Intervention arm showed a 45% higher likelihood of PPFP adoption among women who delivered at home (adjHR 1.45, 95% CI: 1.01-2.07).  No significant difference was observed in PPFP adoption among women who delivered in a facility between intervention and comparison arms. | Integrating PPFP messages into community-level services alongside facility-based care enhances PPFP adoption, particularly among women delivering at home in Ethiopia.  This approach addresses the unmet need for contraception in postpartum women, contributing valuable insights for integrated maternal and child health services in similar contexts. |
| Anagaw, T. F., et al. (2022)[65] | Cross-Sectional Study | Mothers | 624 | Maternity waiting home (MWH) utilisation | Mothers in East Bellessa district, northwest Ethiopia | Maternity waiting home (MWH) utilisation was 20.5% (95% CI=17.3-23.7) | Factors positively associated with MWH use included the husband's educational status, knowledge about MWHs, experiential attitude, perceived behavioural control, and behavioural intention. | Maternity waiting for home utilisation was low, being affected by several factors. |
| Hagedorn, B. L., et al. (2023)[66] | Sub-national analysis | Population in Ethiopia | Not applicable | Clinical workload estimation | Regions and charted cities of Ethiopia | Varies by region | Workload varied significantly between regions, and an increase is projected across all areas due to population growth and changing disease patterns. Sensitivity analysis highlighted the impact of fertility assumptions on workload estimates and the importance of considering local factors in healthcare staffing. | The findings underscore the inadequacy of fixed population ratios for healthcare workforce planning in Ethiopia. Policymakers should tailor staffing allocations based on regional demographics, disease trends, and other local factors to ensure equitable access to care and efficient healthcare delivery systems. |
| Selamu, M., et al. (2019)[67] | Cohort Study | Primary healthcare workers | 145 | Burnout in healthcare workers | Rural primary healthcare facilities | High depression symptoms (aMD 0.56, 95% CI 0.29, 0.83, p < 0.01); Two or more stressful life events (aMD 1.37, 95% CI 0.06, 2.14, p < 0.01);  Community health extension worker vs. facility-based HCW (aMD 5.80, 95% CI 3.21, 8.38, p < 0.01); Perceived job insecurity (aMD 0.73, 95% CI 0.08, 1.38, p = 0.03); Older age (aMD 0.36, 95% CI 0.09, 0.63, p = 0.01) | Burnout in healthcare workers showed a non-significant reduction over six months.  Factors such as high depression symptoms, multiple stressful life events, being a community health extension worker, perceived job insecurity, and older age were significantly associated with higher levels of emotional exhaustion. | There was no significant change in burnout levels in the short term following the addition of mental healthcare duties. Longer-term studies are needed to substantiate these findings. This evidence can inform the development of interventions to enhance well-being and reduce burnout among healthcare workers. |
| Shaw, B., et al. (2015)[68] | Cross-Sectional | Caregivers of sick children | Not specified | - Utilization of health extension workers | Rural health posts in Oromia | 9.3% of caregivers utilised HEWs for a child with diarrhea, fever, and/or pneumonia  There was a higher likelihood of utilisation in iCCM areas (OR: 1.44; 95% CI: 0.97-2.12; P = 0.07), but the effect disappeared after accounting for confounders.  Utilisation is associated with maternal education, illness type, and distance in iCCM areas. Perceptions of illness severity and service quality are the primary reasons for non-utilization. | Low utilisation of HEWs by caregivers of sick children, with only 9.3% using HEWs for their child’s illness.  Maternal education, illness type, and distance were factors influencing utilisation. Perceptions of illness severity and service quality were the primary reasons for non-utilization.  Despite reaching some vulnerable populations, there were significant barriers to the use of BEWs delivering iCCM services. | Demand generation efforts and minimising remaining barriers are urgently needed for the sustained success of the iCCM strategy in Ethiopia. The study highlights the importance of addressing these barriers to improve the utilisation of health extension workers in delivering iCCM services. |
| Tadesse GL., et al. (2021)[69] | Qualitative study and review | 244 health centers | N/A | Policies and delivery models for integrating MH/SA services into primary health care | Ethiopia | In 2020, 243 health centers completed mhGAP training, and 5,000 urban HEWs received refresher training, including mental health and NCDs. | This study highlighted the national integration of MH/SA services into primary health care, prevention, early detection, treatment, and rehabilitation, involving traditional healers and community-based organisations in Ethiopia.  Key factors for success included stakeholder buy-in, task-sharing, and supervision. | The integration of MH/SA services into primary health care in Ethiopia has improved service quality and health outcomes. The experience provides lessons on stakeholder buy-in, gradual integration, and ongoing supervision and mentorship. These lessons are transferable to other countries aiming to integrate MH/SA services into primary health care. |
| Tolera, H., et al. (2020)[70] | Community-based, cross-sectional study | Women in Gida Ayana Woreda, Ethiopia | 454 | Utilisation of antenatal care (ANC) services in rural western Ethiopia | Rural western Ethiopian women | Only 15.2% of women made the recommended minimum number of ANC visits.  Women with fewer than two children were 10.7 times more likely to receive the recommended ANC.  Those receiving two or more home visits by HEWs were 9.7 times more likely to receive the recommended ANC. | Factors associated with ANC utilisation in rural Ethiopia include the number of children, home visits by health extension workers (HEWs), engagement in gainful activities, experience of high fever, and administrative decentralization. | Program interventions are needed to meet WHO recommendations for ANC visits, focus on birth spacing, enhance HEW home visits, improve awareness of pregnancy complications, target socioeconomic development targeting poor households, and decentralise health systems for better ANC accessibility in rural areas of western Ethiopia. |
| Yitayal, M., et al. (2014)[71] | Cross-sectional study | Mothers in Ethiopia | 1320 | Utilisation of Health Extension Program (HEP) and associated factors | Community-based in Ethiopia | Health extension workers visited 52.7% of households frequently, and 78.5% of mothers visited health posts. Factors associated with higher utilisation included frequent household visits by health extension workers, being part of model households, understanding of HEP packages, and higher family income. | The study underscores the importance of frequent home visits by health extension workers and the role of model households in enhancing community utilisation of HEP services. It highlights the need for ongoing training and support for model households and improving community understanding of HEP services. | Recommendations include prioritising continuous home visits for non-model households, ongoing support for existing model households, expanding model-family training to more households, and strengthening communication strategies to improve HEP implementation and utilisation of essential health services in Ethiopia. |
| Asmare, G., et al. (2022)[72] | Cross-sectional study | Children aged 12-23 months in Southwest Ethiopia | 644 | Full immunisation coverage among urban and rural children | Urban and rural areas of Southwest Ethiopia | Overall, full immunisation coverage was 66.1% (74.3% in urban areas, 59.2% in rural areas) with a significant difference. | Urban residence, higher wealth index (OR = 1.82, 95% CI 1.12-2.96), ANC follow-up (OR = 2.05, 95% CI 1.17-3.59), and fear of COVID-19 infection at health institutions (OR = 1.67, 95% CI 1.04-2.67) were predictors of full immunisation.  Knowledge and place of delivery influenced urban areas, while distance (OR = 0.58, 95% CI 0.35-0.97) and male partner involvement (OR = 1.62, 95% CI 1.01-2.61) influenced rural areas. | Vaccination coverage was higher in urban areas than in rural areas, but it is still far below the WHO-recommended target. |
| Asresie, M. B., et al. (2023)[73] | Cross-sectional study | Rural men in Ethiopia | 10,187 | HIV testing uptake among rural men | Rural Ethiopia | Overall, only 40.3% of rural men have ever been tested for HIV | Many risk factors have been identified, and a lack of health insurance coverage (AOR = 0.54, 95% CI [0.42-0.69]) is associated with lower service uptake. | The study highlights the need for targeted awareness programs, integration of HIV testing with healthcare services, and strategies like partner accompany and home-based testing to enhance HIV testing uptake among rural men in Ethiopia. |
| Atalell, K. A., et al. (2022)[74] | Ecological study | Ethiopia | Not applicable | Spatiotemporal distribution and ecological determinants of BCG vaccination coverage | General population in Ethiopia | Ethiopia's national BCG vaccination coverage averaged 65.5% between 2000 and 2019, rising from 53.5% in 2000 to 79.0% in 2019. Despite a 47.6% increase, substantial geographical inequalities persist, with higher coverage in northern, western, and central regions. | Factors like temperature, altitude, and population density positively influenced national BCG vaccination coverage, while healthcare access barriers negatively affected it. | Despite significant overall improvement in BCG vaccination coverage in Ethiopia, persistent geographical disparities highlight the need for targeted interventions to maintain high coverage and effectively prevent tuberculosis. |
| Baye, K., et al. (2022)[75] | Analysis of Ethiopian Demographic and Health Survey data | Women and children in Ethiopia | 8997 | Trends and socioeconomic inequalities in coverage of reproductive, maternal, newborn, and child health (RMNCH) interventions; association with child nutritional outcomes | General population in Ethiopia | Significant increase in national co-coverage index (2005-2019)  Pro-rich and pro-urban distribution of RMNCH interventions (p < 0.05)  Highest inequality observed for skilled assistance during delivery (SII: 80.4%), access to an improved source of drinking water (SII: 62.6%), and antenatal care visits (SII: 55.5%)  Low coverage and inequality in RMNCH associated with stunting, wasting, and minimum dietary diversity (MDD) | Integrating nutrition interventions into existing health interventions shows promise but requires equitable distribution to ensure no one is left behind. Reducing socioeconomic inequality in RMNCH is key to achieving health, nutrition, and equity-related goals. | Addressing socioeconomic inequalities in RMNCH coverage is crucial for improving child nutritional outcomes and achieving the Sustainable Development Goals' health, nutrition, and equity-related goals. |
| Eregata, G. T., et al. (2019)[76] | National and subnational analysis | General population in Ethiopia | Not applicable | Estimation of the 2015 national and subnational UHC service coverage status for Ethiopia | General population in Ethiopia | Overall national UHC service coverage (2015): 34.3%  UHC service coverage in Addis Ababa: 52.2%  UHC service coverage in the Afar region: 10%  Coverage for non-communicable diseases: 35%  Coverage for reproductive, maternal, neonatal and child health: 37.5%  Coverage for infectious diseases: 52.8%  UHC service capacity and access coverage: 20%  Substantial regional variations (e.g., 3.7% in the Somali region to 41.1% in the Harari region) | Ethiopia's 2015 overall UHC service coverage was low compared with most of the other countries in the region. Significant regional disparities were observed, requiring targeted interventions to improve coverage and equity. | Ethiopia should rapidly scale up promotive, preventive, and curative health services by increasing primary healthcare investment. Policymakers should address regional disparities by redistributing the health workforce, improving health resources, and providing focused technical and financial support to low-performing regions. |
| Gebremedhin, A. F., et al. (2023)[77] | Cross-sectional study | Women with their most recent children (aged 12-23 months) | 1891 | Continuum of maternal, newborn, and child health care and associated factors in Ethiopia | Women and children in Ethiopia | Average composite coverage index value: 39%  Overall completion rate of the continuum of care: 2%  4% of women did not receive any services along the continuum of care  Postnatal care for newborns had the lowest coverage: 12% | The study identified several factors that influence the continuum of care, including women's educational status, region, residence, socio-economic status, perceived distance to health facilities, pregnancy intention, mode of delivery, parity, and early antenatal care initiation. The impact of these factors varied across different levels of the composite coverage index. | The findings underscore the need for integrated and targeted strategies to improve the continuum of care in Ethiopia, considering the identified determinants to address maternal and child health service gaps effectively. |
| Gedlu, E., et al. (1997)[78] | Cluster sample survey using EPI methodology | Children aged 12-24 months and mothers | 213 children, 1269 households | Immunisation coverage and problems associated with vaccination delivery in Gondar, Northwest Ethiopia | Children and mothers in Gondar | Fully immunised children: 47.4%  Not immunised at all: 30%  Partially immunised: 22.6%  Mothers receiving tetanus toxoid more than once: 38% | Reasons for incomplete or missed immunisations included lack of knowledge, unawareness of dose schedules and measles protection, preference for the disease over-vaccination, social issues, and lack of time. | Polio coverage among children was low because many were born at home and missed recommended birth immunisations. |
| Gelagay, A. A., et al. (2021)[79] | Cross-sectional study | Mothers with children aged 12-23 months | 584 mothers | Full immunisation coverage and determinants among children aged 12-23 months | Children aged 12-23 months in Wogera district, Northwest Ethiopia | Full immunisation coverage: 76.5% (95% CI 73.2-79.8) | Factors associated with complete immunisation: Mother age >40 years (AOR=7.37, 95% CI: 1.65-32), Women empowerment (AOR=1.57, 95% CI: 1.13-2.39), 1-3 ANC visits (AOR=2.51, 95% CI: 1.14-5.52), 4+ ANC follow-up (AOR=2.73, 95% CI: 1.26-5.91), Health extension worker's home visit during postpartum (AOR=1.76, 95% CI: 1.10-2.84), Male involvement in immunisation (AOR=3.27, 95% CI: 1.84-5.81), Birth order of 6 and above (AOR=0.35, 95% CI: 0.14-0.86) | Maternal health care uptake, women empowerment, home visits by health extension workers, and male involvement were significant predictors of complete vaccination. |
| Haile, T. G., et al. (2024)[80] | Cross-sectional analysis | Children under five years old in Ethiopia | 2096 children | Adequate coverage of curative child health services | Children under five years old in Ethiopia | Crude coverage to curative health service use: 38.4% (95% CI: 36.5-40.4)  Overall quality of care index: 54.4% (weighted from structure 30.0%, process 9.2%, outcome 15.2%)  Adequate coverage: 20.9% (95% CI: 19.9-22.0) | The overall quality of care was moderate, with significant variations in structure, process, and outcome components.  National adequate curative child health services coverage ranges from 16.9% to 34.6% across regions. | If child health-related targets are to be achieved in Ethiopia, system-wide interventions are required to address both demand-side and supply-side bottlenecks in the provision of child health services. |
| Hanlon, C., et al. (2019)[81] | Qualitative situational analysis | People with mental disorders in Ethiopia | Not specified | Moving towards universal health coverage for mental disorders | People with mental disorders in Ethiopia | Not applicable | Significant mental health burden in Ethiopia supported by local epidemiological data.  Political commitment to address mental health issues.  Integration of mental health into primary care. Favourable macro-fiscal environment for health investments. Challenges include low government health expenditure on mental health (estimated at 0.3% of GDP), shortage of specialists, and weak implementation capacity. | Strategies proposed include expanding public health insurance, leveraging resources from SDG programs, and implementing existing plans for task-shared mental health care, alongside advocacy for increased attention to mental health in Ethiopia and beyond. |
| Terefe, B., et al. (2022)[82] | Cross-sectional study | Household members | 8,663 | Spatial distribution and factors of community-based health insurance coverage | Households in Ethiopia | Only 28.1% had health insurance coverage. | The highest health insurance coverage was spatially clustered in Amhara, Tigray, the central part of SNNPR, and some parts of the Oromia and Benishangul regions, while Harari, Somali, Afar, Diredawa, and Gambella areas had the lowest coverage.  Community-based health insurance coverage showed spatial variations across regions in Ethiopia, with clustering in Amhara and Tigray.  Factors influencing coverage included age, education level, wealth index, family size, and region. | Utilisation was significantly lower in regions like Afar, Oromia, Somali, etc., compared to Amhara. |
| Yitbarek et al. (2023)[83] | Assessment using the PHC progression model | Ethiopian PHC system at national, regional, and local levels | Not specified | Capacity assessment in governance, inputs, and population health and facility management domains using a model with 33 measures | Ethiopian PHC system | Governance domain: 2.8 out of 4  Inputs domain: 2.3  Population health and facility management domain: 2 | The governance domain scored 2.8, indicating varying quality in management, priority setting, and innovation.  The inputs domain scored 2.3, highlighting drug, supply, and facility infrastructure management challenges.  The population health and facility management domain scored 2, reflecting moderate capacity.  There is no significant difference in scores between federal and national levels. | Relevant primary healthcare (PHC) policies and leadership exist at the federal and regional levels. However, capacity limitations hinder effective implementation at subnational levels. Challenges with major inputs and data quality further diminish the local PHC system's capabilities. |
| Tassew, B., et al. (2021)[84] | Cross-sectional study | Patients, health service providers, and health facilities | Six health facilities in Addis Ababa | Quality of primary health care during the COVID-19 pandemic | Primary healthcare facilities in Addis Ababa, Ethiopia | Overall patient satisfaction: 77.9%  Health service accessibility: 54.7%  Patient centeredness: 67.9%  Equitability: 72.1%  Timeliness: 63.4% | PHC facilities are characterised by limited ongoing Continuing Professional Development (CPD) opportunities for staff.  Employees' health examination records and in-service education/training are inconsistently recorded.  Half of the facilities lack hand washing rooms, and restroom cleanliness is an issue.  There is poor continuity of care, with only two facilities demonstrating consistent practices. | Most respondents were satisfied with the quality of primary health care services. However, gaps were identified in human resource management, infrastructure, referral system, and continuity of care. Improvement is recommended through guidelines, continuous supervision, mentorship, and training. |
| Abebaw, W. A., et al. (2024)[85] | Cross-sectional study | Women who delivered vaginally | 865 women | Quality of childbirth care along the continuum of care | Women delivering vaginally in Gondar town public health facility, Northwest Ethiopia | Good-quality childbirth care: 59% during admission, 76.8% during intrapartum, 45% during immediate postpartum | Maternal postsecondary educational status and age 25-35 predicted quality of care at admission.  Hospital referrals, the presence of guidelines, and providers aged 25-35 affected the quality of care during intrapartum.  Urban residence, skilled birth attendant experience, and number of delivery couches were associated with quality of care during immediate postpartum. | Improved childbirth care quality compared to previous assessments, but challenges persist, necessitating additional staffing to address service quality issues. Various maternal, provider, and facility factors influence childbirth care quality, highlighting areas for targeted interventions. |
| Ftwi, M., et al. (2020)[86] | Cross-sectional study | Mothers who gave birth before six months | 466 mothers | Completion of four ANC visits based on a recommended schedule | Mothers in Northern Ethiopia | 4+ scheduled ANC completion: 9.9% (95% CI, 7.1-12.4).  63.9% attended 4+ ANC visits regardless of schedule | Factors associated with completion: Community health insurance membership (AOR 2.140), distance from health facility ≤1 hour (AOR 3.921), less household workload (AOR 0.369), husband support (AOR 2.561). | There is a low completion rate of recommended ANC visits in rural Northern Ethiopia. Factors like proximity to health facilities, household workload, and male involvement significantly influence ANC attendance. |
| Gebru, T. et al (2018)[87] | Cross-sectional study | Household heads (CBHI insured and uninsured) in Ethiopia | 1964 (982 CBHI insured, 982 uninsured) | Impact of Community-Based Health Insurance (CBHI) on Health-Related Quality of Life (HRQoL) | Household heads in Ethiopia | HRQoL score: CBHI insured: 63.02, uninsured: 58.92.  Factors influencing HRQoL: marital status, occupation, education level, family size, wealth index.  HRQoL was 12.41% higher among CBHI insured compared to uninsured household heads. | CBHI has a positive impact on HRQoL among insured household heads in Ethiopia.  Factors like marital status, occupation, education level, family size, and wealth index significantly influence HRQoL. | The study demonstrates that CBHI contributes positively to the quality of life among insured household heads in Ethiopia. |
| Kasaye, H. K., et al. (2023)[88] | Cross-sectional study | Women receiving antenatal care | 2,660 | Effects of combining antenatal care visits at health posts and health centers on antenatal care quality | Women in rural Ethiopia | Quality increased by 20% (adjusted IRR= 1.20 [1.12-1.28]) | Quality of antenatal care improved when care was combined at health posts and health centers, compared to receiving care only at health posts.  Socioeconomic status and setting variations influenced care quality. | Combining antenatal care from health posts and health centers enhances care quality in rural Ethiopia. |
| Ketaro, M. K., et al. (2021)[89] | Cross-sectional study | Caretakers in health centers | 411 exit interviews, 60 consultation sessions | Quality of Integrated Management of Newborn and Childhood Illness (IMNCI) services | Caretakers in Jimma, Ethiopia | Mean satisfaction score: 63.4  Dissatisfaction was noted in waiting time (23.4%), explanation received (33.6%), and availability of medicines (19.2%). | Factors significantly associated with satisfaction included the availability of prescribed medications and receiving laboratory services. | Satisfaction levels with IMNCI services were lower than national standards, particularly due to issues like waiting times and medication availability. |
| Negash, W. D., et al. (2022)[90] | Facility-based cross-sectional study | Outpatients attending primary healthcare facilities | 417 participants sampled | Health system responsiveness is measured across autonomy, communication, confidentiality, attention, dignity, choice, and amenities. | Outpatients in primary healthcare facilities | Health system responsiveness between insured (70.74%) and uninsured (29.26%) outpatients. | The negative association between age and responsiveness: OR -1.33 (95% CI: -2.47, -0.19) for 30–39 years; OR -1.66 (95% CI: -3.02, -0.32) for 40–49 years.  Positive associations were found with urban residence: OR +1.33 (95% CI: 0.37, 2.29), perceived quality of healthcare: OR +2.96 (95% CI: 1.95, 4.05), and patient satisfaction: OR +3 (95% CI: 1.94, 4.07). | Health insurance status did not impact health system responsiveness among outpatients. Improvement is needed across all domains of health system responsiveness, especially waiting times and healthcare provider choices. |
| Shiferaw, K., et al. (2021)[91] | Panel study design | Ethiopian women nested within 217 enumeration areas | 2855 respondents | Adequacy and timeliness of antenatal care (ANC) visits | Pregnant women in Ethiopia | Timeliness of ANC initiation: 26.8%, Adequate ANC visits attendance: 43.3% | Factors associated with the timeliness of ANC initiation: rural residence (AOR=0.55, 95% CI: 0.36-0.84), higher education (AOR=2.64, 95% CI: 1.47-4.77), multiparity (AOR=0.53, 95% CI: 0.32-0.89), and partner encouragement (AOR=1.98, 95% CI: 1.14-3.44).  Factors associated with adequacy of ANC visits: Rural residence (AOR=0.20, 95% CI: 0.12-0.35), higher education (AOR=2.96, 95% CI: 1.38-6.15), partner encouragement (AOR=2.11, 95% CI: 1.31-3.40), and timely ANC initiation (AOR=4.59, 95% CI: 2.93-7.21). | Only a quarter of pregnant women they initiated ANC visits in the first trimester, highlighting delays in ANC initiation.  Nearly half attended adequate ANC visits, with disparities across rural and urban settings and socioeconomic backgrounds. |
| Fekadu, W., et al. (2022)[92] | Mixed-method study | People with Severe Mental Health Conditions (SMHCs) and comparisons in Butajira and Sodo districts, Ethiopia | 336 (168 SMHCs, 168 comparisons) | Consequences of COVID-19 on mental health and mental health services | People with SMHCs in rural Ethiopia | Wellbeing: SMHCs 52 vs. comparisons 72 (p<0.001)  Social support: SMHCs 8.68 vs. comparisons 9.29 (p<0.001) - Food insecurity: SMHCs 26.0% vs. comparisons 12.5% (p<0.001) | People with SMHCs experienced significantly lower well-being, reduced social support, worse living standards, and higher food insecurity compared to comparisons.  Increased relapse, exacerbated stigma, and increased use of restraint were reported among people with SMHCs.  Mental healthcare settings saw decreased patient flow but an increase in new cases. Innovations included flexible medication dispensing and longer appointment intervals. | COVID-19 had detrimental effects on people with SMHCs and mental health services in rural Ethiopia. - Adaptive responses during the pandemic may enhance health system resilience and should be considered in future crisis planning. |
| Mihretu, A., et al. (2023)[93] | Mixed methods study | People with mental health conditions, key informants | In-depth interviews with 16 key informants | Impact of COVID-19 on mental health care and people with mental health conditions | People with mental health conditions in Ethiopia | Not specified | Minimal initial response to mental health aspects of COVID-19.  Mental health care settings diverted to COVID-19 treatment centers, leading to poorer quality care for mental health patients.  It increased stigma and discrimination. - Worsening poverty and decreased access to care in communities.  Medication shortages increased relapse and adverse outcomes.  Neglect of mental health needs in quarantine and treatment facilities initially.  Late development of integrated services addressing both physical and mental health needs. | The COVID-19 pandemic significantly worsened the lives of people with mental health conditions in Ethiopia.  Emergency responses should prioritise this vulnerable population's human rights, health, social, and economic needs. |
| Shuka, Z., et al. (2022)[94] | Retrospective health facility survey | Urban population accessing healthcare services | Data from 59 health centers and 29 public hospitals | Use of healthcare services during COVID-19 pandemic, focusing on MCH and adult services | Urban Ethiopia | Sharp reduction in inpatient (20%-27%) and outpatient (27%-34%) care during COVID-19 pandemic | Maternal and child health (MCH) services remained largely unaffected, including skilled birth attendance, immunisation, and postnatal care.  Family planning and antenatal care initially declined (8%-17%) but recovered post-initial impact period.  The use of other adult services such as ART, TB, leprosy, and dental care was also impacted, but the specifics are not detailed. | Despite overall reductions in healthcare use, COVID-19 did not significantly disrupt MCH services in urban Ethiopia.  Proactive measures by government and healthcare facilities mitigated service disruptions, highlighting resilience in a resource-constrained setting.  Further research is needed to understand the effectiveness of strategies to protect essential healthcare services during crises. |
| Yesuf, E. A., et al. (2023)[95] | Mixed-methods survey | Health facilities in Ethiopia (Amhara, Oromia, Sidama, Southern Nations, Nationalities, and People's Region, Dire Dawa) | 452 health facilities surveyed | Delivery status of essential health services during COVID-19 pandemic | Ethiopia | Adoption of essential health services lists: Woredas (districts) 81.4%, Health facilities 51.2%  Nearly all health centers provide antenatal care services.  Availability: Blood pressure measuring apparatus and delivery set (100%), Radiant warmer (50%), Malnutrition services (47%), Functional incinerator (41%)  Provision of cardiovascular disease management (27.2%), HIV/AIDS treatment (43.5%) | Optimal adoption of essential health service lists, but disparities in service availability across different health needs.  High coverage in maternal healthcare services contrasted with lower coverage in neonatal care, malnutrition treatment, and cardiovascular disease management.  Recommendations for district health systems to enhance maintenance and delivery of essential health services. | The adoption of lists of essential health services was optimal. The status of delivery of essential health services was high for maternal healthcare. Neonatal care at birth, malnutrition treatment, and cardiovascular disease management were low. |
| Alebachew, A., et al. (2023)[96] | Costing Study | Health posts, health centers, primary hospitals | 20 health posts, 25 health centers, eight primary hospitals | Total and per capita PHC costs, PHC resource gaps | Nine Ethiopian regions | Based on the HSTP II targets for 2024/25, the overall resource gap ranged from 48 to 87%, with gaps of 53 to 75% in health posts, 39 to 83% in health centers | The total cost of PHC was US$ 11,532 in health posts and US$ 254,340 in health centers.  The average actual PHC cost per capita in nine Ethiopian regions ranged from US$ 4.7 to US$ 20.2 overall, with US$ 1.5 to US$ 2.9 in health posts and US$ 2.6 to US$ 9.4 in health centers with, significantly lower compared to normative cost (US$ 38.5 per capita) needed for high-quality services. | Compared to the normative cost of US$ 38.5 per capita, all these estimates of actual PHC expenditures were significantly lower, indicating a shortfall in the funding required to deliver an expanded package of high-quality services to a larger population in line with GoE targets. |
| Berman, P., et al. (2018)[97] | Projection Model | Ethiopian government | Not applicable | Potential of domestic resource mobilisation compared to future health care costs, sustainability of primary health care system under varying scenarios of external support decline | Ethiopia | Not applicable | If natural external resources remain at 2011 levels, domestic resource mobilisation alone may cover only half of the estimated healthcare delivery costs during Ethiopia's current health sector plan (2016-2020). Sustainability depends on economic growth and increased government funding. | Ethiopia can likely sustain and further develop its primary healthcare system amidst declining external support, but achieving this will require significant economic growth and increased domestic funding. External partners should support Ethiopia's transition to more significant domestic funding to maintain positive health progress. |
| Alemayehu, Y. K., et al. (2023)[98] | Mixed-method study | Households, HEWs, HPs, and HCs | Household survey: 7122, Survey of HEWs: 584, Assessment of HPs: 343, Assessment of HCs: 179, Randomly selected woredas: 62 | Interaction with HEWs, Availability of HPs and HEWs, Skills and motivation of HEWs, Fulfillment of HP standards | Women interacting with HEWs: 54.8%, Men interacting with HEWs: 32.1%, Female youths interacting with HEWs: 21.9%  Certified HEWs: 57.3%, Average satisfaction score of HEWs: 48.6%, HPs fulfilling equipment standards: 5.4% | Critical gaps in skills and motivation of HEWs  Only a small proportion of HPs met equipment standards | This study informed policy and program decisions by the Ministry of Health, contributing to the design of the HEP Optimization Roadmap 2020-2035 and the development of the Health Sector Transformation Plan II. |  |
| Asnake, M., et al. (2020)[99] | Cross-sectional study | Women who gave birth within 12 months prior to the study. | 884 women. | Immediate postpartum family planning use. | Prevalence of immediate postpartum family planning | Women who used maternity waiting homes: 44%  Women who did not use maternity waiting homes: 36%. | Maternity waiting homes significantly increased the immediate uptake of postpartum family planning (OR = 0.69, 95% CI = 0.51-0.95, p<0.022). | Maternity waiting homes significantly improved uptake of immediate postpartum family planning within 10 minutes to 48 hours after delivery. |
| Feyisa, D., et al. (2022)[100] | Cross-sectional study | Vaccine cold chain handlers at primary health facilities | 140 primary health workers from 28 primary health facilities (4 health centers and 24 health posts) | Knowledge of vaccine cold chain management, adherence to WHO vaccine storage codes, and vaccine management practices | Primary health workers | 54% of respondents had satisfactory knowledge of vaccine cold chain management.  71.4% correctly identified the recommended temperature range (2°C - 8°C) for vaccine storage.  63.6% were aware of twice-daily temperature recordings.  46.2% of facilities had poor adherence to WHO storage practice codes.  60% of facilities had undesirable vaccine management practices. | Around two-thirds (63.6%) of them were aware of the twice-daily temperature recordings. Nearly half, (46.2%) of primary healthcare facilities have experienced poor adherence to the WHO storage practice codes. | This study reveals significant gaps in vaccine cold chain management knowledge and adherence to WHO guidelines at primary healthcare facilities, resulting in widespread poor vaccine management practices. Effective collaboration among stakeholders is essential to enhance vaccine cold chain management and ensure the integrity and efficacy of vaccination programs. |
| Mitike, G., et al. (2023)[101] | Qualitative study | Leaders across Ethiopia's 10 regions and 2 administrative cities | 59 key informants | Health system response to COVID-19 | Leaders from 10 regions and 2 administrative cities in Ethiopia | N/A | Health leaders reassigned professionals, added COVID-19 responsibilities, blocked leave, and hired new staff.  Challenges arose in obtaining PPE and supplies.  Community awareness was raised through media and house-to-house education.  Shifted to home-based isolation for mild cases.  Essential health services were initially neglected.  Multisectoral engagement declined as the pandemic progressed. | Local government authorities and health systems across Ethiopia responded early to the pandemic with multisectoral support and resource allocation.  The intensity of the response waned, and essential services suffered as the pandemic progressed. There is a need to learn from the pandemic and invest in health system basics and coordination of interventions. |
| Negash, W. D., et al. (2024)[102] | Cross-sectional study | Mothers in Ethiopia who gave birth in the last two years | 4469 mothers | Quality of intrapartum care and its associated factors | 4469 mothers | Prevalence of quality intrapartum care was 23.8% (95% CI 22.6, 25.13). | Factors related to quality of intrapartum care include primary education, wealthy household class, history of ANC, urban residence, Tigray region, community-level poverty, and having 2-4 Children | Less than one in four mothers received good-quality intrapartum care. |
| Olyaeemanesh, A. et al. (2019)[103] | Cross-sectional study | Health centers and skilled health workers in Afar, Dire-Dawa, and Tigray regions of Ethiopia | N/A | Availability and inequality in accessibility of health centre-based PHC resources | Health centers and skilled health workers in Afar, Dire-Dawa, and Tigray regions of Ethiopia | Median health centers per 15,000 inhabitants: Afar (0.781), Dire-Dawa (0.566), Tigray (0.591). Median skilled health workers per 10,000 inhabitants: Afar (5.250), Dire-Dawa (7.539), Tigray (6.246). | Health centers and skilled health workers were unevenly distributed. Gini indices for health centers: Afar (0.237), Dire-Dawa (0.280), Tigray (0.216). Gini indices for skilled health workers: Afar (0.347), Dire-Dawa (0.186), Tigray (0.175). | Availability and inequalities in PHC resources vary across regions in Ethiopia. |
| Tessema, G. A., et al. (2020)[104] | National surveys | Health facilities (public and private) and FP service users in Ethiopia | 1094 health facilities (139 private, 955 public); 3696 women | Structural quality of FP services and use in public vs. private facilities | Health facilities (public and private) and FP service users in Ethiopia | Only 16% of private and 22% of public PHCUs reported having health providers available 24/7  92% of private facilities charged fees; 99% of public facilities provided FP services for free.  STI services: 35% (public) vs. 90% (private).  FP methods accessed: 3110 (84%) in public, 586 (16%) in private. 82% of rural women accessed FP services from public facilities, while 50% accessed from private facilities. | The private facilities were less likely to have implants (Adjusted Odds Ratio (AOR) = 0.06; 95% Confidence Interval (CI): 0.03, 0.12), trained FP providers (AOR = 0.23; 95% CI: 0.14, 0.41) and FP guidelines/protocols (AOR = 0.33; 95% CI: 0.19, 0.54) than public facilities but were more likely to have functional cell phones (AOR = 8.20; 95% CI: 4.95, 13.59) and water supply (AOR = 3.37; 95% CI: 1.72, 6.59). | The structural quality of FP services in private facilities is lower compared to public facilities in terms of availability of implants, trained providers, and FP guidelines/protocols, but higher in terms of functional cell phones and water supply. |
| Assefa Y et al., (2020)[105] | Macro-analysis | Not specified | Not specified | Relationship between GHS and UHC indices | Not specified | Not specified | There is a moderate and significant relationship between global health security index (GHSI) and universal health cover index (UHCI) (r = 0.662, p<0.001).  There is no relationship between GHSI and the NCDs index (r = 0.063, p>0.05). Negative correlation between GHS threats and capacity for GHS (r = -0.604, p<0.001) and UHC (r = -0.792, p<0.001). | There is inadequate global preparedness for health security, and no country or region is fully prepared for GHS. The aspiration for GHS will not be realized without UHC, which needs a synergistic solution. |
| Syoum, BC et al., (2024)[106] | Qualitative study | Residents of Gojjam, Ethiopia | Not specified | Health infrastructure development and its impact on health security | Gojjam Province, Ethiopia | Not specified | Health infrastructure has a significant impact on health security.  The research highlights the historical development of medical institutions and infrastructure in Gojjam Province, focusing on the equitable allocation of resources and the evolution of health services. The study also examines the types of care centres and health security issues over time. | This research provides insights into the historical growth of healthcare infrastructure and its impact on health security in Gojjam. It aims to help experts, the local community, and policymakers understand the evolution and distribution of health services in the region. |
| Tesema, A. G., et al. (2021)[107] | Qualitative study | National and regional policymakers, officials from partner organizations, woreda/district health office managers, coordinators, and PHC workers | 22 key informants | Explored the capacity and readiness of Ethiopia’s PHC system to deliver integrated, people-centred NCD services | Primary health care facilities in Ethiopia | Not specified | Identified gaps in the WHO Operational Framework levers: under-investment in NCDs, insufficient political commitment, weak governance structures, poor inter-sectoral coordination, and inadequate funding.  Operational issues included fragmented information systems, lack of equipment and medicines, and insufficiently skilled PHC workforce | Strengthening NCD prevention and control through PHC in Ethiopia requires more outstanding political commitment and investment. Success strategies from other PHC programs should be adapted for NCD policies and practices, considering the unique nature of NCDs. |
| Ali, D., et al. (2022)[108] | Case study | Key informants from Private health facilities and public sectors | 50 key informants and 106 health facilities | Assessed Private Health Sector Programs (PHSP) performance in leadership and governance, access to medicines, health management information systems, human resources, service provision, and finance | Private health facilities in Ethiopia | Not specified | Private Health Sector Programs (PHSP) addressed all six WHO health system building blocks.  Challenges include fragile partnerships between public and private bodies, resource constraints, mistrust, limited incentives, and oversight of service quality | The study identified progress in developing an enabling policy environment, engaging PHFs in service delivery through PPM, integrating PHFs in government systems, and building trust through evidence-informed advocacy. |
| Hailu, A., et al. (2021)[109] | Fiscal space analysis | Public health sector in Ethiopia | Not specified | Estimated the financial resources required to implement the Ethiopian Essential Health Services Package (EHSP) from 2020 to 2030 | Ethiopian public health sector | The largest share of costs was for medicines, commodities, and supplies (50-70%), followed by human resources (10-17%). | To implement the EHSP, 13.0 billion USD (94 USD per capita) would be required in 2030. The expected available resources would be 63 USD per capita, creating a 33% gap in resources needed by 2030. | There is a gap between resource needs and expected resources for EHSP implementation. External aid, economic growth, and government commitment are crucial for health funding in Ethiopia. The government and stakeholders must create a reliable mechanism to increase health budgets, improve efficiency, and reduce costs. |
| Fantozzi, P. L., et al. (2021)[110] | Geographic database creation | Health facilities in Gambella Region, Western Ethiopia | 3 primary hospitals, 26 health centers, 121 health posts | Mapped the access roads and location of health facilities using Geomatic Approaches and related technologies | Gambella Region, Western Ethiopia | Not specified | Created a detailed cartographic picture of health facilities' geographical distribution. Network analysis showed the importance of this approach in remodelling a more efficient referral system. | This study demonstrated modern decision-making tools for distributing human and instrumental resources, highlighting the need for efficient referral systems. |

**Reference**

1. Alamneh TS, Teshale AB, Yeshaw Y, Alem AZ, Ayalew HG, Liyew AM, et al. Barriers for health care access affects maternal continuum of care utilization in Ethiopia; spatial analysis and generalized estimating equation. PLoS ONE. 2022;17(4). doi: 10.1371/journal.pone.0266490. PubMed PMID: WOS:000792887700040.

2. Karim AM, Tamire A, Medhanyie AA, Betemariam W. Changes in equity of maternal, newborn, and child health care practices in 115 districts of rural Ethiopia: implications for the health extension program. BMC Pregnancy & Childbirth. 2015;15(1):1-11. doi: 10.1186/s12884-015-0668-z. PubMed PMID: 110194963. Language: English. Entry Date: 20180801. Revision Date: 20180804. Publication Type: Journal Article.

3. Hierink F, Oladeji O, Robins A, Muñiz MF, Ayalew Y, Ray N. A geospatial analysis of accessibility and availability to implement the primary healthcare roadmap in Ethiopia. COMMUNICATIONS MEDICINE. 2023;3(1). doi: 10.1038/s43856-023-00372-z. PubMed PMID: WOS:001080755400001.

4. Tadesse T, Gelaw B, Haile Y, Bisrat F, Kidanne L, Asres M, et al. Immunization service availability and readiness in primary health care in pastoral and semi-pastoral CGPP Ethiopia implementation districts. Ethiopian Journal of Health Development. 2019;33(3). PubMed PMID: WOS:000486967900002.

5. Drown L, Amberbir A, Teklu AM, Zelalem M, Tariku A, Tadesse Y, et al. Reducing the equity gap in under-5 mortality through an innovative community health program in Ethiopia: an implementation research study. BMC Pediatrics. 2024;23(SUPPL 1). doi: 10.1186/s12887-023-04388-1. PubMed PMID: WOS:001175985200001.

6. Fentie EA, Asmamaw DB, Negash WD, Belachew TB, Baykeda TA, Addis B, et al. Spatial distribution and determinants of barriers of health care access among female youths in Ethiopia, a mixed effect and spatial analysis. Scientific reports. 2023;13(1). doi: 10.1038/s41598-023-48473-y. PubMed PMID: WOS:001122989300054.

7. Zimmerman LA, Yi YY, Yihdego M, Abrha S, Shiferaw S, Seme A, et al. Effect of integrating maternal health services and family planning services on postpartum family planning behavior in Ethiopia: results from a longitudinal survey. BMC Public Health. 2019;19(1). doi: 10.1186/s12889-019-7703-3. PubMed PMID: WOS:000496447100006.

8. Tiruneh GT, Zemichael NF, Betemariam WA, Karim AM. Effectiveness of participatory community solutions strategy on improving household and provider health care behaviors and practices: A mixed-method evaluation. PLoS ONE. 2020;15(2) (no pagination).

9. Astale T, Chenault M. Help-Seeking Behavior for Children with Acute Respiratory Infection in Ethiopia: Results from 2011 Ethiopia Demographic and Health Survey. PLoS ONE. 2015;10(11). doi: 10.1371/journal.pone.0142553. PubMed PMID: WOS:000364433100092.

10. Ketema B, Kaba M, Negash S, Addissie A, Kantelhardt EJ. Intention to Undergo Clinical Breast Examination and Its Associated Factors among Women Attending Rural Primary Healthcare Facilities in South Central Ethiopia. Breast Care. 2023;18(6):464-72. doi: 10.1159/000531944. PubMed PMID: 174384858. Language: English. Entry Date: 20231227. Revision Date: 20231227. Publication Type: Journal Article.

11. Abate Y, Solomon K, Azmera YM, de Fouw M, Kaba M. Barrier analysis for continuity of palliative care from health facility to household among adult cancer patients in Addis Ababa, Ethiopia. BMC Palliative Care. 2023;22(1):1-9. doi: 10.1186/s12904-023-01181-w. PubMed PMID: 163718131. Language: English. Entry Date: 20230522. Revision Date: 20230531. Publication Type: Journal Article.

12. Bekele A, Alem A, Seward N, Eshetu T, Gebremariam TH, Getachew Y, et al. Barriers and enablers to improving integrated primary healthcare for noncommunicable diseases and mental health conditions in Ethiopia: a mixed methods study. medRxiv. 2023;22.

13. Endris BS, Fenta E, Getnet Y, Spigt M, Dinant GJ, Gebreyesus SH. Barriers and facilitators to the implementation of nutrition interventions at primary health care units of Ethiopia: A consolidated framework for implementation research. Maternal & Child Nutrition. 2023;19(1):1-12. doi: 10.1111/mcn.13433. PubMed PMID: 160783333. Language: English. Entry Date: 20221222. Revision Date: 20221230. Publication Type: Journal Article.

14. Enyew AM, Dolamo BL. THE CONTRIBUTION OF A HEALTH EXTENSION PROGRAMME IN THE UTILISATION OF MATERNAL HEALTHCARE SERVICES IN THE MECHA DISTRICT OF THE AMHARA REGION, ETHIOPIA. Africa Journal of Nursing & Midwifery. 2017;19(2):1-14. doi: 10.25159/2520-5293/1510. PubMed PMID: 125737023. Language: English. Entry Date: 20171209. Revision Date: 20200123. Publication Type: Journal Article.

15. Aregay A, O'Connor M, Stow J, Ayers N, Lee SS. Palliative care in Ethiopia's rural and regional health care settings: a qualitative study of enabling factors and implementation challenges. BMC Palliative Care. 2023;22(1). doi: 10.1186/s12904-023-01283-5. PubMed PMID: WOS:001086722800001.

16. Aregay A. Perceived policy-related barriers to palliative care implementation: a qualitative descriptive study. Palliative Care and Social Practice. 2023;17. doi: 10.1177/26323524231198542.

17. Gesesew HA, Ward P, Woldemichael K, Lyon P, Mwanri L. Policy and practice suggestions to improve performance on the UNAIDS 90-90-90 targets: Results from a nominal group technique with HIV experts in Southwest Ethiopia. Health Expectations. 2020;23(5):1326-37. doi: 10.1111/hex.13115. PubMed PMID: WOS:000555765000001.

18. Shimels T. The Trend of Health Service Utilization and Challenges Faced During the COVID-19 Pandemic at Primary Units in Addis Ababa: A Mixed-Methods Study. Health Services Research and Managerial Epidemiology. 2021;8. doi: 10.1177/23333928211031119.

19. Argaw MD, Desta BF, Kibret MA, Abebe MG, Heyi WK, Mamo E, et al. Accelerating the performance of district health systems towards achieving UHC via twinning partnerships. BMC Health Services Research. 2020;20(1):N.PAG-N.PAG. doi: 10.1186/s12913-020-05741-1. PubMed PMID: 145996802. Language: English. Entry Date: 20210214. Revision Date: 20210826. Publication Type: Journal Article.

20. Badacho AS, Mahomed OH. Facilitators and barriers to integration of noncommunicable diseases with HIV care at primary health care in Ethiopia: a qualitative analysis using CFIR. Frontiers in public health. 2023;11:1247121.

21. Donovan L, Habte T, Batisso E, Getachew D, Stratil AS, Tekalegne A, et al. Improving neglected tropical disease services and integration into primary healthcare in Southern Nations, Nationalities and People's Region (SNNPR), Ethiopia: results from a mixed methods intervention evaluation. medRxiv. 2023;12.

22. Mayston R, Alem A, Habtamu A, Shibre T, Fekadu A, Hanlon C. Participatory planning of a primary care service for people with severe mental disorders in rural Ethiopia. Health Policy & Planning. 2016;31(3):367-76. doi: 10.1093/heapol/czv072. PubMed PMID: 113537064. Language: English. Entry Date: 20180726. Revision Date: 20190423. Publication Type: Journal Article.

23. Tadesse E, Ekström EC, Berhane Y. Challenges in Implementing the Integrated Community-Based Outpatient Therapeutic Program for Severely Malnourished Children in Rural Southern Ethiopia. Nutrients. 2016;8(5). doi: 10.3390/nu8050251. PubMed PMID: WOS:000378780900012.

24. Datiko DG, Bunte EM, Birrie GB, Kea AZ, Steege R, Taegtmeyer M, et al. Community participation and maternal health service utilization: lessons from the health extension programme in rural southern Ethiopia. Journal of Global Health Reports. 2019;3. doi: 10.29392/joghr.3.e2019027.

25. Curry LA, Alpern R, Webster TR, Byam P, Zerihun A, Tarakeshwar N, et al. Community perspectives on roles and responsibilities for strengthening primary health care in rural Ethiopia. Global Public Health. 2012;7(9):961-73. doi: 10.1080/17441692.2012.686114. PubMed PMID: 104420121. Language: English. Entry Date: 20120928. Revision Date: 20200708. Publication Type: Journal Article.

26. Tolera H, Gebre-Egziabher T, Kloos H. Risk factors for women's non-utilization of decentralized primary health care facilities for postnatal care in rural western Ethiopia. THERAPEUTIC ADVANCES IN REPRODUCTIVE HEALTH. 2020;14. doi: 10.1177/1179558120928340. PubMed PMID: WOS:000545653100001.

27. Tekola B, Mayston R, Eshetu T, Birhane R, Milkias B, Hanlon C, et al. Understandings of depression among community members and primary healthcare attendees in rural Ethiopia: A qualitative study. Transcultural Psychiatry. 2023;60(3):412-27. doi: 10.1177/13634615211064367. PubMed PMID: 171808730. Language: English. Entry Date: 20230919. Revision Date: 20230919. Publication Type: Journal Article.

28. Shiferaw F, Letebo M, Feleke Y, Gelibo T, Getachew T, Defar A, et al. Non-communicable diseases in Ethiopia: policy and strategy gaps in the reduction of behavioral risk factors. Ethiopian Journal of Health Development. 2019;33(4):259-68. PubMed PMID: WOS:000514434000008.

29. Awol M, Edosa D, Jemal K. Spatial pattern and determinants of institutional delivery in Ethiopia: Spatial and multilevel analysis using 2019 Ethiopian demographic and health survey. PLoS ONE. 2023;18(2). doi: 10.1371/journal.pone.0279167. PubMed PMID: WOS:001047063700023.

30. Atnafu A, Kebede A, Misganaw B, Teshome DF, Biks GA, Demissie GD, et al. Determinants of the Continuum of Maternal Healthcare Services in Northwest Ethiopia: Findings from the Primary Health Care Project. Journal of pregnancy. 2020;2020:4318197.

31. Adane B, Fisseha G, Walle G, Yalew M. Factors associated with postnatal care utilization among postpartum women in Ethiopia: a multi-level analysis of the 2016 Ethiopia demographic and health survey. Archives of Public Health. 2020;78(1). doi: 10.1186/s13690-020-00415-0. PubMed PMID: WOS:000528901000001.

32. Memirie ST, Metaferia ZS, Norheim OF, Levin CE, Verguet S, Johansson KA. Household expenditures on pneumonia and diarrhoea treatment in Ethiopia: a facility-based study. BMJ Global Health. 2017;2(1). doi: 10.1136/bmjgh-2016-000166. PubMed PMID: WOS:000408727000009.

33. Girmaw F, Adane E, Kassaw AT, Ashagrie G, Baye T. Willingness to Pay for Social Health Insurance Among Health Care Professionals in North Wollo Zone, Amhara Region, Ethiopia: Mixed Method Study. ClinicoEconomics and Outcomes Research. 2023;15:593-606. doi: 10.2147/CEOR.S421461. PubMed PMID: WOS:001039803600001.

34. Beshah SA, Husain MJ, Dessie GA, Worku A, Negeri MG, Banigbe B, et al. Cost analysis of the WHO-HEARTS program for hypertension control and CVD prevention in primary health facilities in Ethiopia. Public Health in Practice. 2023;6. doi: 10.1016/j.puhip.2023.100423.

35. Assefa Y, Hill PS, Gilks CF, Admassu M, Tesfaye D, Van Damme W. Primary health care contributions to universal health coverage, Ethiopia. Bulletin of the World Health Organization. 2020;98(12):894-905A. doi: 10.2471/BLT.19.248328. PubMed PMID: 147418702. Language: English. Entry Date: 20201210. Revision Date: 20201211. Publication Type: Journal Article.

36. Assebe LF, Dillu D, Tiru G, Johansson KA, Bolongaita S, Chakrabarti A, et al. Financial risks of care seeking for malaria by rural households in Jimma Zone, Oromia Region, Southwest Ethiopia: a cross-sectional study. BMJ Open. 2021;11(12). doi: 10.1136/bmjopen-2021-056162. PubMed PMID: WOS:000737300500013.

37. Arage SM, Daba DB, Dessalegn AY. Organizational commitment of health professionals and associated factors in primary healthcare facilities of Addis Ababa, Ethiopia: A multi-center cross-sectional study. Frontiers in public health. 2022;10:981621.

38. Argaw MD, Desta BF, Muktar SA, Abera WS, Beshir IA, Otoro IA, et al. Comparison of maternal and child health service performances following a leadership, management, and governance intervention in Ethiopia: a propensity score matched analysis. BMC Health Services Research. 2021;21(1):1-9. doi: 10.1186/s12913-021-06873-8. PubMed PMID: 152043054. Language: English. Entry Date: 20210825. Revision Date: 20211122. Publication Type: Journal Article. Journal Subset: Biomedical.

39. Bayou B, Hailu T, Jenberie A, Minalu Y, Tesfamichael T. Transforming primary health care unit service delivery through leadership, management and governance (LMG) training: A field action report from Ethiopia. Ethiopian Journal of Health Development. 2020;34(2):33-41.

40. Berhan Y, Ali M, Tassew A, Nonogaki A. Universal Health Coverage Policy and Progress towards the Attainment of Universal Sexual and Reproductive Health and Rights Services in Ethiopia. Ethiopian journal of health sciences. 2022;32(1):181-200.

41. Chantler T, Karafillakis E, Wodajo S, Demissie SD, Sile B, Mohammed S, et al. 'We All Work Together to Vaccinate the Child': A Formative Evaluation of a Community-Engagement Strategy Aimed at Closing the Immunization Gap in North-West Ethiopia. International Journal of Environmental Research and Public Health. 2018;15(4). doi: 10.3390/ijerph15040667. PubMed PMID: WOS:000434868800106.

42. Desta BF, Abitew A, Beshir IA, Argaw MD, Abdlkader S. Leadership, governance and management for improving district capacity and performance: the case of USAID transform: primary health care. BMC Family Practice. 2020;21(1):1-7. doi: 10.1186/s12875-020-01337-0. PubMed PMID: 147387300. Language: English. Entry Date: 20201208. Revision Date: 20201208. Publication Type: Journal Article.

43. Fetene N, Patel A, Benyam T, Ayde A, Desai MM, Curry L, et al. Experiences of managerial accountability in Ethiopia's primary healthcare system: a qualitative study. BMC Family Practice. 2020;21(1):1-9. doi: 10.1186/s12875-020-01332-5. PubMed PMID: 147410292. Language: English. Entry Date: 20201209. Revision Date: 20201209. Publication Type: Journal Article.

44. Hailemichael A, Belayihun B, Asnake M, Lulu K, Desta BF, Genene L, et al. Referral Service Barriers in Ethiopia: Experiences and perceptions of actors. Ethiopian Journal of Health Development. 2021;35(5):55-62.

45. Heyi WK, Gurmamo EM, Anara AA, Sendeku AG, Refissa A, Yadeta FS, et al. An evaluation of excellence in primary healthcare units after the introduction of a performance management innovation in two regional states of Ethiopia: a facility based comparative study. BMC Health Services Research. 2022;22(1):1-12. doi: 10.1186/s12913-022-07885-8. PubMed PMID: 156190924. Language: English. Entry Date: 20220413. Revision Date: 20231116. Publication Type: Journal Article.

46. Liu L, Desai MM, Benyam T, Fetene N, Ayehu T, Nadew K, et al. An Analysis of Zonal Health Management Capacity and Health System Performance: Ethiopia Primary Healthcare Transformation Initiative. International journal of health policy and management. 2022;11(11):2610-7.

47. Liu L, Desai MM, Fetene N, Ayehu T, Nadew K, Linnander E. District-Level Health Management and Health System Performance: The Ethiopia Primary Healthcare Transformation Initiative. International journal of health policy and management. 2022;11(7):973-80.

48. Abajebel S, Jira C, Beyene W. UTILIZATION OF HEALTH INFORMATION SYSTEM AT DISTRICT LEVEL IN JIMMA ZONE OROMIA REGIONAL STATE, SOUTH WEST ETHIOPIA. Ethiopian journal of health sciences. 2011;21:65-76. PubMed PMID: WOS:000421548800008.

49. Asemahagn MA. Determinants of routine health information utilization at primary healthcare facilities in Western Amhara, Ethiopia. Cogent Medicine. 2017;4(1) (no pagination).

50. Ayele W, Biruk E, Kifle A, Habtamu T, Taye G, Wondarad Y. Patterns of essential health services utilization and routine health information management during Covid-19 pandemic at primary health service delivery point Addis Ababa, Ethiopia. Ethiopian Journal of Health Development. 2021;35:90-7. PubMed PMID: WOS:000670303100012.

51. Bogale TN, Teklehaimanot SM, Debela TF, Enyew DB, Bedada AN, Kebebew SD, et al. Barriers, facilitators and motivators of electronic community health information system use among health workers in Ethiopia. FRONTIERS IN DIGITAL HEALTH. 2023;5. doi: 10.3389/fdgth.2023.1162239. PubMed PMID: WOS:001033163700001.

52. Bramo SS, Desta A, Syedda M. Applying the ICT4H model to understand the challenges for implementing ICT-based health information services in primary healthcare in South Ethiopia. Learning Health Systems. 2023;7(3). doi: 10.1002/lrh2.10360.

53. Hailemariam T, Atnafu A, Gezie LD, Kaasboll JJ, Klein J, Tilahun B. Individual and contextual level enablers and barriers determining electronic community health information system implementation in northwest Ethiopia. BMC Health Services Research. 2023;23(1):644.

54. Kassa A, Matlakala MC. Effectiveness of mHEALTH Application at Primary Health Care to Improve Maternal and New-born Health Services in Rural Ethiopia: Comparative study. medRxiv. 2022;06.

55. Kebede M, Adeba E, Chego M. Evaluation of quality and use of health management information system in primary health care units of east Wollega zone, Oromia regional state, Ethiopia. BMC Medical Informatics & Decision Making. 2020;20(1):1-12. doi: 10.1186/s12911-020-01148-4. PubMed PMID: 143738111. Language: English. Entry Date: 20200615. Revision Date: 20210107. Publication Type: Journal Article. Journal Subset: Biomedical.

56. Tadesse AW, Gurmu KK, Kebede ST, Habtemariam MK. Analyzing efforts to synergize the global health agenda of universal health coverage, health security and health promotion: a case-study from Ethiopia. Globalization and Health. 2021;17(1). doi: 10.1186/s12992-021-00702-7. PubMed PMID: WOS:000646892000003.

57. Bisrat F, Abdissa S, Asres M, Tadesse T, Kidanne L, Asegdew B, et al. Healthcare workers' readiness to provide immunization services at primary health care units in pastoral and semi-pastoral regions in Ethiopia: Core Group Polio Project implementation areas. Ethiopian Journal of Health Development. 2019;33(3). PubMed PMID: WOS:000486967900004.

58. Tekle MG, Wolde HM, Medhin G, Teklu AM, Alemayehu YK, Gebre EG, et al. Understanding the factors affecting attrition and intention to leave of health extension workers: a mixed methods study in Ethiopia. Human Resources for Health. 2022;20(1):1-11. doi: 10.1186/s12960-022-00716-1. PubMed PMID: 155339183. Language: English. Entry Date: 20220222. Revision Date: 20220402. Publication Type: Journal Article.

59. Tsigebrhan R, Fekadu A, Medhin G, Newton CR, Prince MJ, Hanlon C. Performance of primary health care workers in detection of mental disorders comorbid with epilepsy in rural Ethiopia. BMC Family Practice. 2021;22(1):1-10. doi: 10.1186/s12875-021-01551-4. PubMed PMID: 153075643. Language: English. Entry Date: 20211026. Revision Date: 20211027. Publication Type: Journal Article.

60. Badacho AS, Mahomed OH. Sustainability of integrated hypertension and diabetes with HIV care for people living with HIV at primary health care in South Ethiopia: implication for integration. BMC Primary Care. 2023;24(1):1-8. doi: 10.1186/s12875-023-02204-4. PubMed PMID: 173725317. Language: English. Entry Date: 20231123. Revision Date: 20231123. Publication Type: Journal Article.

61. Berhanu A, Alemayehu M, Daka K, Binu W, Suleiman M. Utilization of Integrated Community Case Management of Childhood Illnesses at Health Posts in Southern Ethiopia. PEDIATRIC HEALTH MEDICINE AND THERAPEUTICS. 2020;11:459-67. doi: 10.2147/PHMT.S282698. PubMed PMID: WOS:000598075100001.

62. Bradley H, Bedada A, Tsui A, Brahmbhatt H, Gillespie D, Kidanu A. HIV and family planning service integration and voluntary HIV counselling and testing client composition in Ethiopia. AIDS CARE-PSYCHOLOGICAL AND SOCIO-MEDICAL ASPECTS OF AIDS/HIV. 2008;20(1):61-71. doi: 10.1080/09540120701449112. PubMed PMID: WOS:000252511700008.

63. Gebremedhin LT, Giorgis TW, Gerba H. Policies, delivery models, and lessons learned from integrating mental health and substance abuse services into primary health care in Ethiopia. FASEB BioAdvances. 2021;3(9):694-701. doi: 10.1096/fba.2020-00145. PubMed PMID: WOS:000663064700001.

64. Sitrin D, Jima GH, Pfitzer A, Wondimu C, Belete TW, Pleah T, et al. Effect of integrating postpartum family planning into the health extension program in Ethiopia on postpartum adoption of modern contraception. Journal of Global Health Reports. 2020;4. doi: 10.29392/001c.13511.

65. Anagaw TF, Debela Y, Asresie MB, Wasihun Y, Bogale EK. Maternity waiting Home-use and associated factors among mothers in northwest Ethiopia, The application of the integrated behavioral model. Ethiopian Journal of Health Development. 2022;36(2). PubMed PMID: WOS:000822497900001.

66. Hagedorn BL, Han R, McCarthy KA. One size does not fit all: an application of stochastic modeling to estimating primary healthcare needs in Ethiopia at the sub-national level. BMC Health Services Research. 2023;23(1):1070.

67. Selamu M, Hanlon C, Medhin G, Thornicroft G, Fekadu A. Burnout among primary healthcare workers during implementation of integrated mental healthcare in rural Ethiopia: a cohort study. Human Resources for Health. 2019;17(1):N.PAG-N.PAG. doi: 10.1186/s12960-019-0383-3. PubMed PMID: 137587793. Language: English. Entry Date: 20190722. Revision Date: 20210111. Publication Type: Journal Article.

68. Shaw B, Amouzou A, Miller NP, Tsui AO, Bryce J, Tafesse M, et al. Determinants of Utilization of Health Extension Workers in the Context of Scale-Up of Integrated Community Case Management of Childhood Illnesses in Ethiopia. American Journal of Tropical Medicine and Hygiene. 2015;93(3):636-47. doi: 10.4269/ajtmh.14-0660. PubMed PMID: WOS:000361254900039.

69. Tadesse Gebremedhin L, Giorgis TW, Gerba H. Policies, delivery models, and lessons learned from integrating mental health and substance abuse services into primary health care in Ethiopia. FASEB BioAdvances. 2021;3(9):694-701.

70. Tolera H, Gebre-Egziabher T, Kloos H. Using Andersen's behavioral model of health care utilization in a decentralized program to examine the use of antenatal care in rural western Ethiopia. PLoS ONE. 2020;15(1). doi: 10.1371/journal.pone.0228282. PubMed PMID: WOS:000534603400044.

71. Yitayal M, Berhane Y, Worku A, Kebede Y. Health extension program factors, frequency of household visits and being model households, improved utilization of basic health services in Ethiopia. BMC Health Services Research. 2014;14(1):156-. doi: 10.1186/1472-6963-14-156. PubMed PMID: 103817379. Language: English. Entry Date: 20150123. Revision Date: 20240123. Publication Type: Journal Article.

72. Asmare G, Madalicho M, Sorsa A. Disparities in full immunization coverage among urban and rural children aged 12-23 months in southwest Ethiopia: A comparative cross-sectional study. HUMAN VACCINES & IMMUNOTHERAPEUTICS. 2022;18(6). doi: 10.1080/21645515.2022.2101316. PubMed PMID: WOS:000849581300001.

73. Asresie MB, Worku GT, Bekele YA. HIV Testing Uptake Among Ethiopian Rural Men: Evidence from 2016 Ethiopian Demography and Health Survey Data. HIV AIDS-RESEARCH AND PALLIATIVE CARE. 2023;15:225-34. doi: 10.2147/HIV.S409152. PubMed PMID: WOS:000986238500001.

74. Atalell KA, Alemayehu MA, Teshager NW, Belay GM, Alemu TG, Anlay DZ, et al. Mapping BCG vaccination coverage in Ethiopia between 2000 and 2019. BMC Infectious Diseases. 2022;22(1). doi: 10.1186/s12879-022-07547-4. PubMed PMID: WOS:000815063000001.

75. Baye K, Laillou A, Chitekwe S. Co-coverage of reproductive, maternal, newborn and child health interventions shows wide inequalities and is associated with child nutritional outcomes in Ethiopia (2005-2019). Maternal and Child Nutrition. 2022. doi: 10.1111/mcn.13452. PubMed PMID: WOS:000877361000001.

76. Eregata GT, Hailu A, Memirie ST, Norheim OF. Measuring progress towards universal health coverage: National and subnational analysis in Ethiopia. BMJ Global Health. 2019;4(6). doi: 10.1136/bmjgh-2019-001843.

77. Gebremedhin AF, Dawson A, Hayen A. Determinants of continuum of care for maternal, newborn, and child health services in Ethiopia: Analysis of the modified composite coverage index using a quantile regression approach. PLoS ONE. 2023;18(1). doi: 10.1371/journal.pone.0280629. PubMed PMID: WOS:000951825100001.

78. Gedlu E, Tesemma T. Immunization coverage and identification of problems associated with vaccination delivery in Gondar, North West Ethiopia. East African Medical Journal. 1997;74(4):239-41.

79. Gelagay AA, Geremew AB, Teklu A, Mekonnen ZA, Gera R, Ba-Nguz A, et al. Full immunization coverage and its determinants among children aged 12-23 months in Wogera district, Northwest Ethiopia. Ethiopian Journal of Health Development. 2021;35(3):16-27. PubMed PMID: WOS:000729948300004.

80. Haile TG, Benova L, Mirkuzie AH, Asefa A. Effective coverage of curative child health services in Ethiopia: analysis of the Demographic and Health Survey and Service Provision Assessment survey. BMJ Open. 2024;14(2):e077856. doi: 10.1136/bmjopen-2023-077856. PubMed PMID: 38382958.

81. Hanlon C, Alem A, Lund C, Hailemariam D, Assefa E, Giorgis TW, et al. Moving towards universal health coverage for mental disorders in Ethiopia. International Journal of Mental Health Systems. 2019;13(1):N.PAG-N.PAG. doi: 10.1186/s13033-019-0268-9.

82. Terefe B, Alemu TG, Techane MA, Wubneh CA, Assimamaw NT, Belay GM, et al. Spatial distribution and associated factors of community based health insurance coverage in Ethiopia: further analysis of Ethiopian demography and health survey, 2019. BMC Public Health. 2022;22(1). doi: 10.1186/s12889-022-13950-y. PubMed PMID: WOS:000838649800010.

83. Yitbarek K, Serawit A, Medhin G, Alemayehu YK, Teklu AM, Assefa Y, et al. Capacity of the Ethiopian primary health care system to achieve universal health coverage: a primary health care progression approach. Health Policy & Planning. 2023;38(4):474-85. doi: 10.1093/heapol/czad013. PubMed PMID: 163024088. Language: English. Entry Date: In Process. Revision Date: 20240430. Publication Type: Journal Article. Journal Subset: Biomedical.

84. Tassew B, Nega A, Asseffa D, Biruk E, Habtamu T, Taye G, et al. Quality of Primary Health Care during COVID-19 Pandemic in Addis Ababa Ethiopia: Patients-side and facility level assessment. Ethiopian Journal of Health Development. 2021;35(Special Issue 1):98-107.

85. Abebaw WA, Wolde HF, Tilahun WM, Gebreegziabher ZA, Teshome DF. Quality of childbirth care and its determinants along the continuum of care among pregnant women who gave birth vaginally in Gondar town public health facility, Northwest Ethiopia, 2022: generalised structural equation modelling. BMJ Open. 2024;14(4):e073199. doi: 10.1136/bmjopen-2023-073199. PubMed PMID: 38580371.

86. Ftwi M, Gebretsadik GGE, Berhe H, Haftu M, Gebremariam G, Tesfau YB. Coverage of completion of four ANC visits based on recommended time schedule in Northern Ethiopia: A community-based cross-sectional study design. PLoS ONE. 2020;15(8). doi: 10.1371/journal.pone.0236965. PubMed PMID: WOS:000563451300028.

87. Gebru T, Lentiro K. The impact of community-based health insurance on health-related quality of life and associated factors in Ethiopia: a comparative cross-sectional study. Health and Quality of Life Outcomes. 2018;16. doi: 10.1186/s12955-018-0946-3. PubMed PMID: WOS:000434058600002.

88. Kasaye HK, Dadi TL, Yilma MT, Jebena MG, Medhin G, Kassie GM, et al. Does Combining Antenatal Care Visits at Health Posts and Health Centers Improve Antenatal Care Quality in Rural Ethiopia? Ethiopian journal of health sciences. 2023;33(1):37-48.

89. Ketaro MK, Muhammed AH, Abdi AA. Quality of Integrated Management of Newborn and Childhood Illness Services at Health Centers in Jimma, Southwest Ethiopia. PATIENT PREFERENCE AND ADHERENCE. 2021;15:793-805. doi: 10.2147/PPA.S280004. PubMed PMID: WOS:000641386400001.

90. Negash WD, Atnafu A, Asmamaw DB, Tsehay CT. Does Health System Responsiveness Differ between Insured and Uninsured Outpatients in Primary Health Care Facilities in Asagirt District, Ethiopia? A Cross-Sectional Study. Advances in Public Health. 2022:1-10. doi: 10.1155/2022/3857873. PubMed PMID: 160251916. Language: English. Entry Date: 20221226. Revision Date: 20221226. Publication Type: Journal Article.

91. Shiferaw K, Mengistie B, Gobena T, Dheresa M, Seme A. Adequacy and timeliness of antenatal care visits among Ethiopian women: a community-based panel study. BMJ Open. 2021;11(12):e053357. doi: 10.1136/bmjopen-2021-053357. PubMed PMID: 34949623.

92. Fekadu W, Shewangizaw S, Girma E, Fekadu A, Hanlon C. Consequences of COVID-19 on access and delivery of mental health care in two rural Ethiopian districts. A mixed method study. Ethiopian Medical Journal. 2022;60:66-74.

93. Mihretu A, Fekadu W, Alemu AA, Amare B, Assefa D, Misganaw E, et al. Impact of the COVID-19 pandemic on mental health care and people with mental health conditions in Ethiopia: the MASC mixed-methods study. International Journal of Mental Health Systems. 2023;17(1). doi: 10.1186/s13033-023-00612-8. PubMed PMID: WOS:001114669900001.

94. Shuka Z, Mebratie A, Alemu G, Rieger M, Bedi AS. Use of healthcare services during the COVID-19 pandemic in urban Ethiopia: evidence from retrospective health facility survey data. BMJ Open. 2022;12(2). doi: 10.1136/bmjopen-2021-056745. PubMed PMID: WOS:000760940500017.

95. Yesuf EA, Abdisa B, Sime H, Alemu EK, Asseffa NA, Jisso M, et al. Essential Health Services Delivery Status During COVID-19 Pandemic in Ethiopia: A National Mixed-Methods Survey of Primary Healthcare Units. Ethiopian journal of health sciences. 2023;33(2):87-94.

96. Alebachew A, Abdella E, Abera S, Dessie E, Mesele T, Mitiku W, et al. Costs and resource needs for primary health care in Ethiopia: evidence to inform planning and budgeting for universal health coverage. Frontiers in public health. 2023;11:1242314.

97. Berman P, Mann C, Ricculli ML. Can Ethiopia Finance the Continued Development of Its Primary Health Care System If External Resources Decline? Health systems and reform. 2018;4(3):227-38.

98. Alemayehu YK, Medhin G, Teklu AM. National Assessment of the Health Extension Program in Ethiopia: Study Protocol and Key Outputs. Ethiop J Health Sci. 2023;33(Spec Iss 1):3-14. doi: 10.4314/ejhs.v33i1.2S. PubMed PMID: 38362474.

99. Asnake M, Belayihun B, Tilahun Y, Zerihun H, Tasissa A, Tilahun Z, et al. Leveraging maternity waiting homes to increase the uptake of immediate postpartum family planning in primary health care facilities in Ethiopia. Ethiopian Journal of Health Development. 2020;35(1):1-9. PubMed PMID: WOS:000626331900001.

100. Feyisa D, Ejeta F, Aferu T, Kebede O. Adherence to WHO vaccine storage codes and vaccine cold chain management practices at primary healthcare facilities in Dalocha District of Silt'e Zone, Ethiopia. Tropical Diseases, Travel Medicine and Vaccines. 2022;8(1) (no pagination).

101. Mitike G, Nigatu F, Wolka E, Defar A, Tessema M, Nigussie T. Health system response to COVID-19 among primary health care units in Ethiopia: A qualitative study. PLoS ONE. 2023;18(2 February) (no pagination).

102. Negash WD, Asmamaw DB, Wassie GT, Azene AG, Eshetu HB, Terefe B, et al. Less than one in four mothers get quality intrapartum health care services in Ethiopia. Scientific reports. 2024;14(1). doi: 10.1038/s41598-024-54506-x. PubMed PMID: WOS:001173675500095.

103. Olyaeemanesh A, Woldemichael A, Takian A, Sari AA. Availability and inequality in accessibility of health centre-based primary healthcare in Ethiopia. PLoS ONE. 2019;14(3) (no pagination).

104. Tessema GA, Mahmood MA, Gomersall JS, Assefa Y, Zemedu TG, Kifle M, et al. Structural quality of services and use of family planning services in primary health care facilities in Ethiopia. How do public and private facilities compare? International Journal of Environmental Research and Public Health. 2020;17(12):1-11.

105. Assefa Y, Hill PS, Gilks CF, Damme WV, Pas RV, Woldeyohannes S, et al. Global health security and universal health coverage: Understanding convergences and divergences for a synergistic response. PLoS One. 2020;15(12):e0244555. Epub 2020/12/31. doi: 10.1371/journal.pone.0244555. PubMed PMID: 33378383; PubMed Central PMCID: PMCPMC7773202.

106. Syoum BC, Tefera GA. Health infrastructure development and its impact on health security in Ethiopia since the 20th Century: Focus on Gojjam Province. Cogent Arts & Humanities. 2024;11(1):2286063. doi: 10.1080/23311983.2023.2286063.

107. Tesema AG, Abimbola S, Mulugeta A, Ajisegiri WS, Narasimhan P, Joshi R, et al. Health system capacity and readiness for delivery of integrated non-communicable disease services in primary health care: A qualitative analysis of the Ethiopian experience. PLOS Global Public Health. 2021;1(10):e0000026. doi: 10.1371/journal.pgph.0000026.

108. Ali D, Woldegiorgis AG, Tilaye M, Yilma Y, Berhane HY, Tewahido D, et al. Integrating private health facilities in government-led health systems: a case study of the public-private mix approach in Ethiopia. BMC Health Serv Res. 2022;22(1):1477. Epub 2022/12/04. doi: 10.1186/s12913-022-08769-7. PubMed PMID: 36463163; PubMed Central PMCID: PMCPMC9719643.

109. Hailu A, Eregata GT, Stenberg K, Norheim OF. Is Universal Health Coverage Affordable? Estimated Costs and Fiscal Space Analysis for the Ethiopian Essential Health Services Package. Health Syst Reform. 2021;7(1):e1870061. Epub 2021/03/20. doi: 10.1080/23288604.2020.1870061. PubMed PMID: 33739233.

110. Fantozzi PL, Baracca G, Manenti F, Putoto G. Measuring Physical access to primary health care facilities in Gambella Region (Western Ethiopia). Proc Int Cartogr Assoc. 2021;4:30. doi: 10.5194/ica-proc-4-30-2021.
