# Supplementary material for: Strengthening primary health care in Ethiopia: A scoping review of successes, challenges, and pathways towards universal health coverage using the WHO monitoring framework
Source: PLOS Glob Public Health. 2025 Apr 17;5(4):e0004470. doi: 10.1371/journal.pgph.0004470 (PMC12005562; doi:10.1371/journal.pgph.0004470)
Supplement: S3 Table — (DOCX) [file pgph.0004470.s003.docx]

S3 Table. The Arksey and O'Malley methodological framework for conducting a scoping study

| **Arksey and O'Malley Framework Stage** | **Description** |
| --- | --- |
| 1: Identifying the research question | Identifying the research question provides the roadmap for subsequent stages. Relevant aspects of the question must be clearly defined as they have ramifications for search strategies. Research questions are broad in nature as they seek to provide breadth of coverage. |
| 2: Identifying relevant studies | This stage involves identifying the relevant studies and developing a decision plan for where to search, which terms to use, which sources are to be searched, time span, and language. Comprehensiveness and breadth are important in the search. Sources include electronic databases, reference lists, hand searching of key journals, and organizations and conferences. Breadth is important; however, the practicalities of the search are as well. Time, budget and personnel resources are potential limiting factors, and decisions need to be made upfront about how these will impact the search. |
| 3: Study selection | Study selection involves *post hoc*inclusion and exclusion criteria. These criteria are based on the specifics of the research question and on new familiarity with the subject matter through reading the studies. |
| 4: Charting the data | A data-charting form is developed and used to extract data from each study. A 'narrative review' or 'descriptive analytical' method is used to extract contextual or process-oriented information from each study. |
| 5: Collating, summarizing, and reporting results | An analytic framework or thematic construction is used to provide an overview of the breadth of the literature but not a synthesis. A numerical analysis of the extent and nature of studies using tables and charts is presented. A thematic analysis is then presented. Clarity and consistency are required when reporting results. |
| 6: Consultation (optional) | Provides opportunities for consumer and stakeholder involvement to suggest additional references and provide insights beyond those in the literature. |
