# Supplementary material for: Strengthening primary health care in Ethiopia: A scoping review of successes, challenges, and pathways towards universal health coverage using the WHO monitoring framework
Source: PLOS Glob Public Health. 2025 Apr 17;5(4):e0004470. doi: 10.1371/journal.pgph.0004470 (PMC12005562; doi:10.1371/journal.pgph.0004470)
Supplement: Attachment — Submitted filename: Review of Manuscript # PGPH-D-24-02538.pdf [file pgph.0004470.s006.pdf]

PLOS GLOBAL PUBLIC HEALTH

REVIEWER'S COMMENT

Manuscript component	Reviewer's Comment and Suggestion for improvement
**Manuscript Title**	The title is clear and informative. It conveys the primary focus of the manuscript, which is a scoping review of primary health care (PHC) in Ethiopia, with a focus on successes, challenges, and pathways toward achieving Universal Health Coverage (UHC) and health security. It also specifies the use of the WHO's PHC monitoring framework in reviewing relevant studies, which adds credibility and specificity to the methodology.<br><br>However, the title could be slightly overwhelming due to its length and complexity. The multiple concepts it introduces—primary health care (PHC), successes, challenges, pathways, universal health coverage (UHC), health security, and the WHO framework—make it dense. Simplifying the title or breaking it into more focused elements could enhance readability and clarity, helping readers quickly grasp the core focus of the manuscript. Additionally, the inclusion of both UHC and health security may be seen as broad, potentially requiring more specific focus to align with the scoping review's primary aim.<br><br>**Suggestions for improvement:** To improve the clarity and readability of the title, it would be beneficial to streamline the content by focusing on the central themes and eliminating redundancy. Breaking the title into more focused elements will help readers immediately grasp the main subject without being overwhelmed. Additionally, narrowing the scope to either "Universal Health Coverage" or "Health Security" would allow for a more precise focus, given that both are broad and may not be fully addressed in a single review. A more concise version could emphasize the key components of the scoping review, such as Ethiopia's PHC system and its alignment with global health goals.<br><br>**Suggested title:**<br>"Strengthening Primary Health Care in Ethiopia: A Scoping Review of Successes, Challenges, and Pathways Towards Universal Health Coverage Using the WHO's PHC Monitoring Framework"
**Abstract**	The abstract presents a clear and comprehensive overview of the study, which evaluates Ethiopia's primary health care (PHC) system using the World Health Organization's (WHO) PHC Monitoring Framework. The alignment of the study with global health goals, particularly Universal Health Coverage (UHC) and the Sustainable Development Goals (SDGs), is highly relevant and timely. The manuscript showcases significant strengths, notably the authors' rigorous review of a wide range of relevant articles utilizing the Arksey & O'Malley Methodological Framework by

	systematic searching five major databases and hand-searched relevant articles on Ethiopian PHC. The inclusion of diverse studies, such as national surveys, qualitative studies, mixed-method studies and others, enhances the validity of the findings. Additionally, the abstract effectively highlights both the successes of Ethiopia's PHC system, such as improved healthcare access and leadership commitment, as well as the challenges it faces, providing a balanced perspective on the country's health system. The conclusion highlights the importance of tackling systemic challenges by leveraging past successes to enhance the resilience of primary healthcare, align it more closely with the health needs of the population, and ultimately achieve universal health coverage in Ethiopia.<br>The scoping review method is appropriate for summarizing existing literature, but it does not offer in-depth analysis of individual studies or data, which could lead to missed opportunities for uncovering deeper insights, particularly in the context of regional disparities or specific health system weaknesses.<br><br>While stage six of the Arksey & O'Malley methodology is optional, consulting relevant stakeholders could enrich the findings by providing qualitative insights into challenges The lack of stakeholder perspectives in the abstract, such as insights from healthcare providers, patients, or policymakers, is another notable gap. Incorporating these voices could have added more understanding of the real-world challenges and successes of Ethiopia's PHC system.<br><br>**Suggestions for Improvement:** To enhance the study's depth and applicability, it would be beneficial to incorporate a broader analysis beyond the WHO PHC Monitoring Framework, exploring additional frameworks or local adaptations that might better capture Ethiopia's unique health system challenges. Expanding the scope to include local innovations or practices that have shown promise in addressing health system gaps could also enrich the findings. Further, while the scoping review methodology is useful for summarizing literature, the study could benefit from a more critical analysis of the included studies to identify patterns, correlations, or emerging trends that could guide policy recommendations. The abstract would also benefit from more concrete suggestions for overcoming the identified challenges, such as strategies for improving resource allocation, addressing rural inequities, and fostering stronger governance and service integration. Finally, offering policy recommendations based on the findings could provide practical guidance for decision-makers in Ethiopia to effectively address the challenges and build on the successes of the PHC system.

**Introduction**	The introduction provides a clear and thorough background on Ethiopia's primary health care (PHC) system, effectively situating it within the context of the country's socio-political, economic, and developmental landscape. The historical overview of Ethiopia's adoption and evolution of PHC, from a curative-oriented approach to a more inclusive, community-focused strategy, is well articulated and highlights the significant progress made. The inclusion of Ethiopia's Health Sector Transformation Plans (HSTP) and emphasis on multisectoral collaboration demonstrates a well-rounded understanding of the factors driving the country's success in expanding PHC and moving towards Universal Health Coverage (UHC) by implementing a national flagship program, the health extension program. The introduction also effectively frames the importance of strategic partnerships, community engagement, and political commitment in supporting the growth of Ethiopia's PHC system. Furthermore, the identification of gaps in existing studies and the articulation of the need for a structured framework-based approach underscore the relevance and importance of the proposed scoping review.<br><br>While the introduction presents a comprehensive narrative, there are a few gaps and limitations that could be addressed to strengthen the overall argument. One limitation is the reliance on broad historical and policy-focused statements without a deep dive into specific challenges or current obstacles in the PHC system, particularly in underserved areas. While the text mentions the achievements and efforts made, it does not sufficiently explore the ongoing systemic or infrastructural challenges in rural or remote areas, where the PHC system is still facing significant limitations. Additionally, while the focus on multisectoral collaboration is emphasized, the introduction does not provide specific examples of successful or failed multisectoral partnerships, which could offer more practical insights into the complexities of these collaborations. Another gap is the brief mention of the health extension program; the introduction could benefit from a more detailed exploration of how HEP has contributed to improving health outcomes, particularly in rural areas, including roadmap to optimize the health extension program.<br><br>**Suggestions for Improvement:** To enhance the introduction, it would be beneficial to incorporate more specific details about the ongoing challenges Ethiopia faces in its PHC system, especially in terms of regional disparities, resource allocation, and workforce distribution being impacted by narrow fiscal space. These aspects would provide a more balanced view of both the successes and challenges within the system. Additionally, a more in-depth discussion of the health extension program and its impact on improving health access to basic health services and outcomes in underserved areas would strengthen the narrative and provide concrete examples of Ethiopia's PHC approach in practice.

	Including case studies or specific instances of multisectoral collaboration, both successful and challenging, would offer readers a more understanding of how these partnerships work in practice and their impacts.
**Methods**	The methodology section is clearly structured, outlining the systematic approach to the scoping review and adhering to the Arksey and O'Malley framework from stage 1 to 5, which is widely recognized for its rigorous and transparent process. The inclusion of five well-known databases (PubMed, Scopus, Embase, Web of Science, CINAHL) ensures comprehensive coverage of the relevant literature, while the detailed description of the search strategy, including the use of Boolean operators and database-specific adaptations, demonstrates methodological rigor. The dual screening process for study selection, involving multiple authors and consensus resolution, enhances the reliability of the study selection. Additionally, the use of the WHO PHC monitoring conceptual framework to guide the identification of successes and challenges in Ethiopia's PHC system adds a well-established and logical framework for organizing the findings. The incorporation of thematic synthesis and adherence to PRISMA-ScR guidelines further strengthens the review's methodological credibility.<br><br>The exclusion of grey literature, such as reports from local government bodies or non-governmental organizations, may limit the scope of the review and exclude valuable context-specific insights that are often overlooked in academic publications. The limited scope of studies published in English could also introduce language bias, especially considering Ethiopia's multilingual context. Moreover, the reliance on two authors for data extraction and checking may not be sufficient to ensure consistency in a large review, and involving a larger team in this process could further improve reliability.<br><br>**Suggestions for Improvement:** To improve the clarity and robustness of the methodology, expanding the inclusion criteria to include grey literature, such as government reports, policy documents, and publications from development partners or non-governmental organizations, could enrich the review by providing context-specific insights that are often omitted from peer-reviewed studies. To minimize potential language bias, it might be worth considering the inclusion of studies published in other languages, such as Amharic, or providing a justification for excluding non-English studies if this is not feasible. Additionally, involving more authors in the data extraction and verification process would enhance the reliability of the results and reduce the potential for bias or error. Finally, providing a clearer description of the thematic analysis process, including how themes were identified and the specific subthemes used for categorization, would help

	readers understand the analytical framework and ensure transparency in the reporting of results.
**Result**	**Strengths:** The Results section of the manuscript provides a comprehensive and detailed analysis of the primary health care (PHC) system in Ethiopia, structured around key themes relevant to achieving Universal Health Coverage (UHC). The inclusion of a large number of studies (110 studies) across various research types, including cross-sectional, qualitative, mixed-method studies, and other studies, is a significant strength. This method offers a comprehensive and detailed view of the advancements and obstacles facing Ethiopia's primary healthcare system. The use of the WHO PHC Monitoring Framework to structure the findings is also a notable strength, providing a clear and systematic way to assess the PHC system's progress. Furthermore, the identification of various domains such as structure of the PHC system, inputs, service delivery process, and outcomes adds depth to the review, covering the multifaceted nature of the primary health care system. The synthesis of findings into thematic categories—governance, adjustments to population health needs, financing, physical infrastructure, health workforce, health information systems, digital health, and more—helps readers to easily grasp the areas where Ethiopia has made progress and where further attention is required. The presentation of findings with evidence from numerous studies contributes to the transparency and rigor of the review.<br><br>**Gaps/Limitations:** One of the key limitations of the Results section is the lack of clarity in some areas due to the over-reliance on vague phrases such as "Error! Reference source not found." These placeholders, while possibly indicating areas that were meant to be populated with figures or references, detract from the overall professionalism of the manuscript and can confuse the reader. Additionally, while the review is thorough in its discussion of challenges and barriers, some sections are overly generalized, such as the mention of the "lack of priority and inadequate commitment" for certain services like non-communicable diseases (NCDs) and palliative care into PHC system. These might be due to lack of adequate financial and material resources on top of inadequately trained workforce. It would be beneficial to elaborate on specific examples, case studies, or quantitative data to substantiate these claims. Furthermore, the section on digital health is relatively limited in scope, particularly given the increasing global interest in digital health technologies. It would be helpful to expand this section by addressing specific examples of successful (sustained, not related specific projects) mHealth interventions or digital health innovations implemented in Ethiopia, alongside the challenges and barriers to digital health. While the inclusion of studies on health workforce issues is valuable, the results

section could benefit from a more structured approach to these findings, such as grouping them by specific workforce challenges (e.g., staff turnover, training gaps, geographic disparities) to make the narrative clearer. The review identified many challenges and a few strengths of the primary health care system while there are many promising developments. The manuscript particularly highlighted significant shortcomings in areas such as adjustment to population health needs, primary health care financing, physical infrastructure, health workforce quality, health information systems, and other related domains, which predominantly emphasized weaknesses. This focus on deficiencies may stem from a critical review of existing studies that primarily concentrate on the challenges facing PHC systems

Over the past two to three decades, Ethiopia has significantly advanced its primary health care system through the expansion of pre-service training programs for health cadres, such as health extension workers, midwives, health officers and other midlevel professionals. These developments have collectively contributed to better health outcomes, with marked improvements in key health indicators across the country.

While hospitals are traditionally associated with secondary and tertiary care, they also play a crucial role in delivering essential primary health care services, including chronic disease management, mental health support, and integrated care pathways. The role of hospitals in delivering integrated primary health care services is overlooked in the scoping review, which can hinder efforts to develop holistic health policies and practices that truly reflect the continuity of care patients require.

Suggestions for Improvement:

1. **Addressing Placeholder References**: The manuscript should ensure that all references (e.g., “Error! Reference source not found”) are properly populated with relevant figures, tables, or citations to maintain the quality and professionalism of the manuscript.
2. **Clarification and Elaboration on Key Findings**: It would enhance the clarity and impact of the results to provide more detailed, concrete examples, especially in the discussion of challenges like the low integration of NCD and palliative care services. Specific case studies or quantitative data would strengthen these claims and offer readers a clearer understanding of the situation.
3. **Expansion on Digital Health**: Given the increasing importance of digital technologies in healthcare, the section on digital health should be expanded to provide more in-depth analysis. Discussing successful mHealth interventions, their impact on health

	outcomes, and the barriers to digital health adoption would make this section more robust. Addressing the role of digital health in improving service delivery during the COVID-19 pandemic is particularly relevant and could be expanded.<br>4. **Structuring Health Workforce Findings**: The section on the health workforce could be more structured by categorizing the challenges into distinct themes such as staff shortages, turnover, burnout, and training gaps, as well as successes in health workforce development and deployment. This would improve readability and allow for a more systematic discussion of the issue.<br>5. **Strengthen Analysis on Health Security**: There is a noted gap in the analysis of health security in the PHC system, which could be addressed more explicitly. Expanding on the relationship between health security and PHC, including potential risks and the integration of health security measures within the PHC framework, would be valuable for readers concerned with health system resilience.<br>6. **Provide Clearer Data on Service Coverage and Inequality**: The results section briefly touches on service coverage and inequality but lacks a more detailed breakdown. Adding figures or examples to illustrate the extent of disparities in access to PHC services across different regions, urban vs. rural areas, and among different population groups would further underscore the gaps and provide more actionable insights.<br>7. **Improving Transitions between Sections**: The review could benefit from smoother transitions between themes, ensuring that the findings related to each topic are logically connected and flow coherently from one section to the next. This would improve readability and help the reader easily follow the narrative.
**Discussion**	The discussion section of this manuscript provides a thorough and insightful review of Ethiopia's primary healthcare (PHC) system, offering a comprehensive analysis of successes and challenges in achieving universal health coverage (UHC) and health security. The use of the WHO's PHC performance monitoring framework provides a robust structure that aligns the findings with global health priorities, offering valuable context for policymakers and researchers. The inclusion of specific case examples, such as the integration of maternal health services and family planning and the innovative health extension program, is particularly strong, as it illustrates the practical application of strategies and highlights Ethiopia’s progress in key areas. The paper also effectively integrates the concept of community empowerment, illustrating how local participation in healthcare can drive better health outcomes and further UHC goals. The discussion of multisectoral collaboration, despite its

	challenges, is well-articulated, providing a nuanced understanding of its critical role in strengthening PHC.<br><br>**Gaps/Limitations:** While the discussion is comprehensive, it would benefit from a deeper exploration of the limitations and challenges faced by Ethiopia in scaling up the successes identified. For example, although the paper mentions governance and political commitment issues at regional and district levels, it would be beneficial to elaborate on the specific political or structural barriers that hinder multisectoral collaboration and the integration of services. Additionally, while the manuscript mentions gaps in health infrastructure, digital health, and ICT, these aspects could be explored in greater detail, particularly regarding the long-term sustainability of these systems. Furthermore, while the discussion acknowledges that further empirical research is needed, it could provide a clearer call to action for future studies, particularly those focused on local-level data or areas where evidence is scarce, such as the resilience of the PHC system during crises like war or natural disasters. The exclusion of non-English publications could also be more thoroughly addressed to explain how this may limit the generalizability of the findings.<br><br>**Suggestions for Improvement:** To strengthen this discussion section, it is recommended to include more detailed examples of the political and governance challenges at various levels of the healthcare system, particularly focusing on how regional and district-level leadership can be more effectively engaged in the implementation of PHC policies. Additionally, expanding the analysis of the barriers to service integration, particularly related to infrastructure and human resources, would provide a more holistic understanding of the challenges Ethiopia faces in expanding and sustaining PHC services. Given the importance of digital health and ICT in the future of PHC, a more detailed exploration of Ethiopia's ICT challenges—such as internet access, data quality, and technology adoption—would be useful. Finally, the manuscript could benefit from a more explicit connection between the identified challenges and potential solutions, such as recommending specific actions or policy adjustments that could address the governance issues, improve infrastructure, and enhance multisectoral coordination. Expanding the call for empirical research to include underexplored topics like the impact of service integration on PHC system resilience in emergencies, and detailing how to engage the broader community in sustaining PHC, would help make the paper even more actionable for future research and policy development.
**Conclusion**	The conclusion section effectively summarizes the key points discussed in the manuscript, offering a balanced assessment of Ethiopia's progress toward achieving universal health coverage (UHC) through its primary

	healthcare (PHC) system. Integration of services like family planning, HIV, and mental health, provides a clear indication of the system's successes. The review has acknowledged that robust governance and political commitment, particularly through the national health extension program, have significantly improved access to services and strengthened community engagement. Furthermore, the discussion of challenges such as the inadequate prioritization of non-communicable diseases (NCDs), palliative care, uneven allocation of resources, and infrastructure shortcomings is pertinent and reflects a deeper comprehension of the complex healthcare landscape. The conclusion highlights practical solutions, including focused investments, governance reforms, and the enhancement of multisectoral policies, providing important insights for policymakers and researchers. Additionally, the recommendation to empower communities and invest in the training of local leaders is both compelling and forward-thinking.<br><br>**Gaps/Limitations:** While the conclusion highlights critical challenges and suggests important areas for improvement, it could benefit from more specific examples or actionable details regarding how these challenges might be overcome. For example, the mention of "targeted investments" and "governance reforms" could be expanded to include more concrete recommendations or specific policy approaches. The lack of detail on how to address financial constraints and weak multisectoral collaboration leaves these challenges somewhat vague, making it harder for the reader to envision practical steps. Additionally, while community empowerment and local leadership training are emphasized, the specific roles of these initiatives and their impact on PHC system improvement could be more clearly defined. As community empowerment is key for improved PHC service uptake, its sustainability needs to be given due emphasis. The conclusion also does not discuss the role of evidence-based research or data collection in addressing these challenges, an area that could further strengthen the manuscript's argument for sustained improvements in PHC. Despite identifying numerous shortcomings under the primary health care domains, the recommendations advocate for building on existing strengths within the system. This apparent disconnect highlights a need for a more balanced approach that not only addresses the highlighted shortcomings but also recognizes and leverages the strengths that can facilitate improvements in PHC delivery and outcomes.<br><br>**Suggestions for Improvement:** To enhance the conclusion section, it would be helpful to provide more specific, actionable recommendations for addressing the highlighted challenges. For instance, the manuscript could offer examples of successful governance reforms or multisectoral collaborations from other countries that could be adapted to the Ethiopian context. Concrete strategies for improving financial governance, such as

	detailed steps to enhance funding allocation or innovative funding models for rural health services, could be included. The mention of NCDs, palliative care, and infrastructure gaps should be expanded upon with more concrete policy suggestions, such as prioritizing these areas in health planning and resource allocation. Additionally, the role of evidence-based decision-making in overcoming these challenges could be incorporated, perhaps suggesting the need for strengthened data collection and monitoring systems to guide policy adjustments. Lastly, while the empowerment of communities and local leaders is an important point, the manuscript could be improved by offering more details on how these efforts can be scaled, sustained and integrated into the broader PHC system, along with examples of effective community engagement models. By adding these specifics, the conclusion would offer a more detailed roadmap for future progress in Ethiopia's PHC system.
**References**	***Strengths in the References/ Referencing:***<br><br>• **Diversity and Relevance**: The references listed cover a wide range of topics that align well with the manuscript's focus on primary health care (PHC) and universal health coverage (UHC) in Ethiopia. The citations reflect important areas such as health system strengthening, community-based health insurance, the health extension program, and the role of leadership and governance in improving PHC services. These topics are all highly relevant to the paper's content and contribute to the depth and scope of the manuscript.<br>• **Timeliness and Coverage of Recent Literature**: The reference list includes many recent publications (e.g., from 2020 to 2024), ensuring that the manuscript is grounded in the most up-to-date research in the field. These references incorporate key studies on the impacts of PHC systems, UHC, and health outcomes in Ethiopia, ensuring the manuscript reflects current trends and developments in healthcare research.<br>• **Comprehensive Approach**: The references span a broad range of topics, including COVID-19's impact on healthcare delivery, health system governance, and mental health, which strengthens the manuscript's foundation. This diversity situates the paper within a wider scholarly conversation about Ethiopian health policy, interventions, and healthcare system challenges.<br>• **Use of Reputable and Relevant Sources**: The inclusion of high-quality, reputable sources such as *PLOS ONE*, *BMC Public Health*, *Frontiers in Public Health*, and *Health Expectations* ensures that the references are drawn from well-respected

academic journals. This enhances the manuscript's credibility and situates it within authoritative scholarly research.

Gaps and Limitations:

- **Duplication of Citations**: There is a notable duplication of references, particularly with the *Assefa et al.* (2020) study being cited multiple times (References 5, 11, and 13). This redundancy could confuse readers, making it unclear whether these references refer to the same or different studies. Each reference should be cited only once, and if multiple works from the same author are cited, they should be clearly differentiated using proper citation styles.
- **Missing Citations**: Some references listed in the reference section do not appear to be cited in the text. For example, the reference by *Lavers (2021)* (Reference 2) is not mentioned within the in-text citations, even though it is a key source regarding universal health coverage in Ethiopia. Similarly, studies like *Tadesse et al. (2021)* (Reference 14) and *Verguet et al. (2021)* (Reference 19) may also lack corresponding in-text citations. The author should ensure that every reference listed is properly cited in the text and that all cited studies are included in the reference section.
- **Inconsistent Formatting**: There are inconsistencies in the formatting of references. For example, *References 1 and 36* list the publisher or organization in a somewhat inconsistent manner (e.g., "World Health Organization" versus "WHO" without clarification). A standardized format for listing publishers or organizations should be adopted throughout the reference section to ensure consistency.
- **Lack of Volume and Issue Numbers**: Several references are missing volume or issue numbers, such as *Reference 86 for Aregay et al. (2023)*. This inconsistency may detract from the professionalism of the reference list and could make it more difficult to locate the sources.
- **Inconsistent Capitalization and Journal Name Formatting**: The referencing style lacks consistency in terms of capitalization and the presentation of journal names. For example, some journals have their names fully capitalized, such as *HEALTH EXPECTATIONS* (Reference 88). This formatting issue should be corrected to adhere to a standardized style, ensuring that journal names are presented consistently.
- **Use of Preprints**: Some references listed in the manuscript are preprints (e.g., medRxiv for References 100 and 101). While preprints are useful for sharing emerging research, they are not peer-reviewed articles. To enhance the credibility of the

manuscript, it may be more beneficial to prioritize citing peer-reviewed articles, especially in a journal like *PLOS Global Public Health*, which typically values rigorously peer-reviewed sources. If preprints are necessary, they should be clearly marked as such.

- **Non-English References**: Some references, such as *Kloos (1998)* (Reference 4) and *Negash et al. (2024)* (Reference 27), contain titles in English but may be from journals or sources primarily published in other languages. If applicable, the manuscript should clarify whether translations are available or if these references should be cited in their original language, according to the journal's citation guidelines.

Suggestions for Improvement:

1. **Consolidate Duplicated Citations**: The author should ensure that each study is listed only once in the references list. For instance, if the same study by *Assefa et al.* (2020) is cited multiple times, it should be merged into one entry. Additionally, when multiple works from the same author group are cited, they should be clearly differentiated, either by adding different co-authors or specifying the specific study when needed.
2. **Ensure All References Are Cited in Text**: The author should cross-check the reference list with the manuscript's in-text citations to ensure that every reference is properly cited in the text. Any references not mentioned in the manuscript should be removed from the reference list, and any uncited references in the text should be added to the manuscript. This will maintain clarity and proper attribution of sources.
3. **Ensure Consistent Formatting**: A thorough review of the reference formatting should be conducted to ensure consistency. Each entry should include the necessary components (authors, title, journal, volume/issue, page numbers, and publisher information), adhering strictly to the PLOS Global Public Health style guide. This includes italicizing journal names and correctly formatting volume and issue numbers.
4. **Clarify Language and Source Details**: If any sources are non-English or translations, they should be clearly identified in the reference list. The names of publishers or organizations should follow a standardized format throughout. Additionally, older works, such as *Kloos (1998)*, should be checked for accurate context and translations to ensure they are correctly represented.
5. **Consistent Citation Style**: The formatting of the references section should be standardized according to the PLOS Global Public Health style guide. This includes ensuring that journal

	names are italicized, article titles follow sentence case (not all uppercase), and volume numbers, issue numbers, and page ranges are consistently included where applicable.<br>6. **Double-Check In-Text Citations**: The author should verify that each reference in the list corresponds to an in-text citation. Cross-checking the in-text citations against the reference list is essential to maintain clarity, ensuring that every listed article is referenced appropriately in the manuscript.<br>7. **Remove Redundant Citations**: The author should eliminate or combine duplicate references. For example, the same article by *Gebremedhin et al.* (2021) is listed twice (Reference 94 and Reference 98). Citing it only once would improve efficiency and maintain professionalism in the references section.<br>8. **Reconsider Preprints**: While preprints are valuable for disseminating new research, it may be more appropriate to cite peer-reviewed sources in a manuscript intended for a journal like PLOS Global Public Health. If preprints must be cited, they should be clearly marked as such. Whenever possible, the author should replace preprints with their peer-reviewed versions when they become available.<br>9. **Check Availability**: The author should verify that all references are available and complete in the reference list. Some references may be missing article identifiers (like DOI numbers), or the citation might be incomplete. Ensuring that DOI numbers are included for articles would enhance their accessibility and traceability, contributing to the manuscript's overall quality.<br>10. **Formatting Titles**: Adjust the capitalization of article titles in the references list to maintain consistency. Titles that are entirely in uppercase, such as in Reference 97, should be changed to sentence case to comply with proper formatting conventions for academic journal references.<br><br>By addressing these points, the author will enhance the quality and professionalism of the references section, improving its alignment with PLOS Global Public Health's submission guidelines.

General Comment:

The manuscript titled **"Strengthening Primary Health Care in Ethiopia: A Scoping Review of the Successes, Challenges, and Pathways Towards Universal Health Coverage and Health Security Using the WHO's PHC Monitoring Framework"** provides a well-structured and insightful exploration of the primary health care (PHC) system in Ethiopia. It offers a thorough review of the successes, challenges, and potential pathways toward achieving universal health

coverage (UHC) and health security, applying the World Health Organization's PHC Monitoring Framework as a lens for analysis.

The manuscript's strengths lie in its comprehensive approach, focusing on a highly relevant and pressing issue in global health. It draws on a broad array of studies, with a particular emphasis on Ethiopia's health system challenges and achievements, which is an essential contribution to the field of public health. However, there are areas that need further attention, particularly in reference to clarity, consistency, and citation practices.

Section-by-Section Suggestions:

1. Title:

- **Strengths**: The title is descriptive, clear, and accurately reflects the manuscript's content. It effectively communicates the focus on Ethiopia's PHC and the use of the WHO's PHC Monitoring Framework.
- **Suggestion**: The title is appropriate, but it could be slightly streamlined by reducing redundancy. For instance, "Health Security" may be integrated into the "Pathways Towards Universal Health Coverage" for clarity. A possible revision could be: *"Strengthening Primary Health Care in Ethiopia: A Scoping Review of Successes, Challenges, and Pathways to Universal Health Coverage Using the WHO's PHC Monitoring Framework."*

2. Abstract:

- **Strengths**: The abstract provides a concise overview of the manuscript's aims, methodology, and findings. It summarizes the key themes effectively and provides readers with a clear picture of what the manuscript entails.
- **Suggestions**:
    - Clarify the findings in a more structured manner. For instance, briefly outline the successes, challenges, and pathways mentioned in the results.
    - Explicitly mention the application of the WHO's PHC Monitoring Framework in guiding the scoping review process.
- **Revised Suggestion**: Expand on the abstract by briefly highlighting the critical issues (successes, challenges, pathways) and a sentence on the paper's contribution to policy recommendations or practical insights.

3. Introduction:

- **Strengths**: The introduction effectively sets the stage by emphasizing the importance of strengthening primary health care in Ethiopia. It clearly outlines the problem and the context, focusing on the need for UHC and health security.
- **Suggestions**:

    - Provide a more robust rationale for using the WHO's PHC Monitoring Framework specifically in the Ethiopian context. Explaining why this framework is crucial for the current review would add clarity.
    - A brief summary of the main successes and challenges that have been documented in prior research might help to better situate the study.
- **Revised Suggestion**: Refine the introduction by expanding on the methodology and scope of the scoping review, and offering a clearer connection to the specific goals of strengthening PHC in Ethiopia.

4. Results:

- **Strengths**: The results section appears to provide a solid summary of the key successes and challenges in strengthening PHC in Ethiopia.
- **Suggestions**:
    - Ensure that the results are presented in a clear, organized structure that separates the successes, challenges, and pathways towards UHC.
    - Quantitative data or specific examples from studies could enhance the clarity of the findings and strengthen the evidence.
- **Revised Suggestion**: Add subheadings or bullet points to break the results into distinct sections (e.g., "Successes," "Challenges," "Pathways to UHC"). Ensure any trends or patterns are clearly identified.

5. Discussion:

- **Strengths**: The discussion contextualizes the results and explores the implications of the findings for policy and practice, which is essential for a scoping review.
- **Suggestions**:
    - Clarify the policy implications derived from the findings. It would be beneficial to highlight how the results could influence future health policy or guide improvements in the Ethiopian health system.
    - Provide a comparison with other countries that have similar challenges or have successfully implemented UHC or PHC reforms. This would help the manuscript align with global trends and provide insights for international readers.
- **Revised Suggestion**: Expand the discussion to include potential policy recommendations or insights for Ethiopia, based on the results. A brief comparison with other low-income countries could enrich the discussion.

6. Conclusion:

- **Strengths**: The conclusion reinforces the manuscript's main arguments, emphasizing the need for systemic improvements in Ethiopia's PHC system.
- **Suggestions**:
    - The conclusion could be strengthened by offering more concrete recommendations for future research or policy changes.

  - It might also be helpful to explicitly reference the WHO's PHC Monitoring Framework again as a guiding tool for future action.
- **Revised Suggestion**: Clarify actionable steps that can be taken, based on the scoping review findings, and provide a strong closing statement about the importance of the PHC framework in achieving UHC in Ethiopia.

7. References:

- **Strengths**: The references are comprehensive, drawing on recent and relevant studies in the field of PHC and UHC, particularly for Ethiopia. The inclusion of reputable journals adds credibility to the manuscript.
- **Suggestions**:
  - **Consistency**: Ensure that the reference list follows the *PLOS Global Public Health* citation style meticulously. There are issues with missing volume numbers, inconsistent capitalization, and unnecessary duplications of citations (e.g., Assefa et al. 2020).
  - **Duplication**: Remove duplicate references, such as the multiple citations of the same study (e.g., Assefa et al., 2020) to streamline the references section.
  - **Preprints**: Consider replacing or clarifying the preprints with their peer-reviewed counterparts if possible, or mark them clearly as preprints in line with the journal's guidelines.
  - **In-text Citation Check**: Cross-check the references in the text with the reference list to ensure consistency and completeness.
- **Revised Suggestion**: Revise the references section by adhering strictly to *PLOS Global Public Health* citation guidelines and eliminating inconsistencies or duplications.

Decision

Based on the strengths outlined above and considering the areas for improvement, I would recommend a decision of **Minor Revisions** for the manuscript.

The manuscript demonstrates a strong conceptual foundation and offers valuable insights into Ethiopia's PHC system. However, the author should revise the manuscript to address the issues in referencing, citation consistency, and clarity in some sections. Once these revisions are made, the manuscript would be well-suited for publication in *PLOS Global Public Health*.
