# Supplementary material for: Strengthening primary health care in Ethiopia: A scoping review of successes, challenges, and pathways towards universal health coverage using the WHO monitoring framework
Source: PLOS Glob Public Health. 2025 Apr 17;5(4):e0004470. doi: 10.1371/journal.pgph.0004470 (PMC12005562; doi:10.1371/journal.pgph.0004470)
Supplement: Attachment — Submitted filename: Review of PLOS manuscript Number PGPH-D-24-02538.docx [file pgph.0004470.s005.docx]

Strengthening Primary Health Care in Ethiopia: A Scoping Review of the Successes, Challenges, and Pathways Towards Universal Health Coverage and Health Security Using the WHO’s PHC Monitoring Framework

**General Comment:**

The manuscript should include background that explain the country’s context. Brief introduction about PHC monitoring framework to explain its relevance to evaluate successes and challenges towards UHC and health security.

The material reviewed could be considered relevant for understanding the general situation of PHC system implementation in Ethiopia. The review has also tried to highlight crucial issues on moving forward with the UHC agenda in Ethiopia. Such as:

- Lack of a progress in monitoring system,
- The need for strengthening integrated service delivery,
- Challenges related to governance and political commitment at lower governance level
- The need to invest in the resilience of health systems.

However, there are limited evidences obtained through this review with regards to studies conducted using important indictors suggested by WHO’s PHC monitoring framework, particularly concerning multisectoral action and empowerment of people and communities,

**Methods:**

In this review authors cannot claim they have strictly used the WHO’s PHC Monitoring Framework. The review could be taken as an assessment of the general situation related to Primary Health care implementation towards achieving UHC by reviewing available evidences and help in identifying information gaps. And also the classification of the studies retrieved should have been based on the indicators recommended in the WHO’s PHC Monitoring Framework. The classification made on the type of study does not indicate that the study of success ad challenges is based on the WHO framework specifically. Hence the need to modify the title.

There are two most important indicators used to monitor progress towards UHC i.e service coverage index (SCI) and financial risk protection. Important document I recommend is: Tracking Universal Health Coverage in the WHO African Region, 2022 WORLD HEALTH ORGANIZATION REGIONAL OFFICE FOR AFRICA BRAZZAVILLE — 2022. Also (Primary Health Care on the Road to Universal Health Coverage 2019 GLOBAL MONITORING REPORT). But this review excluded global studies and these important documents are missed. Otherwise it should be realized that global studies are useful for comparison and could initiate exchange of experiences between countries.

**Results:**

Studies Retrieved:

Altogether, 110 studies were included in the review. Some were handpicked.

As suggested above it would have been more relevant if characteristics of the studies have been classified based on their relevance to the important indicators provided by the WHO PHC Monitoring framework.

**Governance:**

There is increasing focus on organizing service delivery at the district level. Accordingly, *Ethiopia’s Woreda or District level transformation* agenda has emphasized the need to create Primary Health Care Unite (PHCU) comprising five satellite HPs, referral HCs and primary hospital with activities planned and monitored at District /Woreda level. The study Team do not seem to follow this definition of PHCU.

**In any case it is important to monitor progress and to find out the successes and challenges of implementing this extremely important initiative that would be necessary for strengthening continuum of care and the referral system. Hence I would like to recommend inclusion of review of studies conducted in relation to district level management, such as that of AMREF: I.E (Woreda Level Deep Dive Assessment to Inform Integrated Health System Strengthening (IHSS) Investment, MERQ Consultancy PLC in Collaboration with Amref Health Africa in Ethiopia, June 2024 Addis Ababa, Ethiopia).** Districts or subnational units play important role in universal health coverage in coordinating service delivery and ensure quality through supportive supervision.

**Specific needs of the population**:

Most of the papers reviewed described important areas of PHC services but very few studies present findings of investigations that describe the actual situation in the country. It is noted that six studies described the importance of tailoring healthcare services and interventions to meet the specific health needs of a population. Yes, but where is the evidence with regards to the actual situation in Ethiopia in this regard?

**Discussion:**

The review was able to highlight primary strategic focus of the health system to achieve UHC. But lacked specific evidences to show the situation in the country due to shortage of focused studies based on WHO’s PHC monitoring framework and specifically to guide implementation of important initiatives.

- The paper has rightly emphasized the need for multisectoral approach to meet the dynamic population health needs. This study illustrates that multisectoral engagement and public-private partnerships within PHC systems remain fragmented and inconsistent. Only two papers looked into this issue and they are not designed to evaluate the contribution of the approach. Presenting country’s specific experience such as the Health Sector Development Program (HSDP) a brief review of Ethiopia’s Sequota Declaration which could serve as exemplary strategy for mulisecoral approach if implemented at scale.
- The importance of community participation is noted. From my own past experiences working in a program that supported strengthening of the HEP whose primary focus has been on creating access to primary health care services. It improved equity and quality of health care through utilization of essential health services, and building community ownership particularly in empowering women, as evidenced by achieving most of the health-related Millennium Development Goals (MDGs) has been great achievement of Ethiopia. But the sustainability of successes in this area needs properly structured monitoring and continued external reviews.
- The statement such as “ The strength of this scoping review is that it provided a **detailed** landscape of PHC in Ethiopia by highlighting the successes, challenges, and pathways towards UHC and health security using the WHO’s PHC Monitoring Framework” cannot be accepted. Limitations in finding relevant studies needs to be emphasized.
- The statement … “Results from this study provide a comprehensive and structured understanding for the researcher, policymakers and program experts to identify, research, and resolve the challenges of achieving UHC and health security”. This is acceptable with the limitation of the review well explained.
- Clear recommendations are expected from the authors. There is a need to highlight areas that require in-depth studies: Such as the monitoring of the implementation of Woreda/District level transformation agenda, a very important initiative to strengthen Primary Health Care Unit establishment and functioning.
- As it could be understood from browsing the references listed in this paper there is lack of periodic assessment of the PHC system particularly to determine capacity of PHC for implementing UHC in Ethiopia. One of the most relevant study cited is reference number 81 (Eregata GT, Hailu A, Memirie ST, Norheim OF. Measuring progress towards universal health coverage: National and subnational analysis in Ethiopia. BMJ Global Health 2019). In this paper it is stated that *“… Few national and subnational studies monitor UHC in low-income countries and there is none for Ethiopia”.*
- Concerning service integration, a need for political commitment and investment at all health system levels for Strengthening NCD prevention and control through PHC in Ethiopia requires emphasis as part of the recommendation.

**Conclusion:**

It is noted that the objective of this review is to evaluate success and challenges towards achieving UHC by using WHO’s PHC Monitoring Framework. However, the finding is that studies designed to monitor the progress underway to ensure the implementation of relevant and attractive health polices is almost non-existent.

However, the reviewers should be appreciated for have coming up with extensive review that describes what needs to be undertaken to implement a strong PHC services and also the need for monitoring system and stress that there is serious lack of focused studies that could help in evaluating success and challenges towards achieving UHC.

There therefore there is a need to revise the presentation of this manuscript to show to Policy Makers and health partners the need for establishing a strong monitoring system by stressing the scarcity of data based on WHO’s PHC Monitoring Framework. Objective of the review should then be to evaluate availability of published studies to review the Successes, Challenges, and Pathways Towards Universal Health Coverage and Health Security Using the WHO’s PHC Monitoring.

Ethiopia’s experience in regularly reviewing HSDP by using external review till the end should have been maintained. It should be remembered that it has been the review of HSDP I that initiated the Health Extension Program (HEP) and subsequent reviews helped in strengthening its implementation.
