# Supplementary material for: Strengthening primary health care in Ethiopia: A scoping review of successes, challenges, and pathways towards universal health coverage using the WHO monitoring framework
Source: PLOS Glob Public Health. 2025 Apr 17;5(4):e0004470. doi: 10.1371/journal.pgph.0004470 (PMC12005562; doi:10.1371/journal.pgph.0004470)
Supplement: Attachment — Submitted filename: Response to Reviewers Feb 24 2025.docx [file pgph.0004470.s007.docx]

**Subject: Revised Manuscript Submission – Response to Feedback**

Dear Reviewers,

I hope this message finds you well. On behalf of all the authors, I would like to extend our sincere gratitude for the opportunity to revise the manuscript and for the time and effort you dedicated to providing invaluable feedback. Your thoughtful and constructive comments have greatly contributed to enhancing the quality and clarity of our work. We have carefully reviewed and addressed each of your suggestions, and we believe that the revised manuscript now provides a more comprehensive and robust portrayal of Ethiopia’s Primary Health Care (PHC) system.

In response to your insightful feedback, we have made several important revisions. We have highlighted the evidence gap regarding multi-sectoral collaboration and community empowerment in achieving Universal Health Coverage (UHC), emphasized governance and leadership issues, and aligned our findings with the WHO Monitoring Framework. Additionally, we expanded our discussion on challenges such as workforce issues, service integration, and barriers to digital health. We also included your valuable suggestion to focus on the strengthening of Non-Communicable Disease (NCD) prevention and control through PHC, which we believe is critical in the context of Ethiopia’s evolving health landscape.

We trust that these revisions strengthen the manuscript and make it more practical and relevant for policymakers and researchers in the field. We have attached the revised manuscript along with a marked-up version indicating the changes made. Once again, we sincerely appreciate your time and constructive feedback, and we look forward to your response to the revised manuscript.

Kind regards,

Tesfaye Mengistu, PhD

On behalf of the authors

**Point-by-Point Response to Reviewer Comments**

| **S. No** | **Reviewer's/Editor's questions/suggestions/comments** | **Authors response** |
| --- | --- | --- |
|  | **Reviewer 1** |  |
| 1. | Please review your reference list to ensure that it is complete and correct. If you have cited papers that have been retracted, please include the rationale for doing so in the manuscript text or remove these references and replace them with relevant current references. Any changes to the reference list should be mentioned in the rebuttal letter that accompanies your revised manuscript.  If you need to cite a retracted article, indicate the article's retracted status in the References list and include a citation and full reference for the retraction notice. | Thanks for the suggestion. All reference lists were checked, and duplicates were removed.  Regarding the inclusion of retracted papers, as far as the author's knowledge is concerned, we did not include any retracted citations. |
| 2. | There is limited evidence obtained through this review with regards to studies conducted using important indicators suggested by WHO's PHC monitoring framework, particularly concerning multi-sectoral action and empowerment of people and communities. | We agree with your observation about the limited evidence related to this issue.  Hence, we have emphasised the evidence gap and highlighted the need for more studies that explore the role of multi-sectoral collaboration and community empowerment in achieving UHC in the abstract and discussion sections. |
| 3. | In addition, the manuscript should include background that explains the country's context, and brief introduction about PHC monitoring framework to explain its relevance to evaluate successes and challenges towards UHC and health security. | Thank you for your suggestion.  In line with the reviewer's suggestions, we included a paragraph regarding the challenges with the currently existing evaluation and monitoring frameworks in the introduction. However, regarding the inclusion of background information, we believe the manuscript adequately captures the core context of Ethiopia's PHC system. |
|  | **Methods:** |  |
| 1. | In this review, authors cannot claim they have strictly used the WHO's PHC Monitoring Framework. The review could be taken as an assessment of the general situation related to Primary Health care implementation towards achieving UHC by reviewing available evidence and help in identifying information gaps. In addition, the classification of the studies retrieved should have been based on the indicators recommended in the WHO's PHC Monitoring Framework. The classification made on the type of study does not indicate that the study of success ad challenges is based on the WHO framework specifically. Hence, the need to modify the title. | Thank you for your feedback.  While the studies included in this review were not initially designed to assess the success and challenges of Ethiopian PHC based on the WHO Monitoring Framework, we extracted the successes and challenges identified within those studies in alignment with the framework's key indicators. We used the WHO framework to organise and present these findings, ensuring the analysis was framed within the framework's context (Figure 2). This approach allowed us to synthesise the available evidence to reflect the framework's guiding principles |
| 2. | There are two most important indicators used to monitor progress towards UHC i.e., service coverage index (SCI) and financial risk protection. Important document I recommend is: Tracking Universal Health Coverage in the WHO African Region, 2022 WORLD HEALTH ORGANISATION REGIONAL OFFICE FOR AFRICA BRAZZAVILLE — 2022. Also (Primary Health Care on the Road to Universal Health Coverage 2019 GLOBAL MONITORING REPORT). This review also excluded global studies and important documents that would be useful for comparison and for exchanging experiences between countries. | Thank you for the valuable suggestions and references.  We have included these documents to compare and provide a broader context in our discussion regarding SCI and financial risk protection indicators. |
|  | **Results:** |  |
| 1. | In terms of studies retrieved, it would have been more relevant if the characteristics of the studies had been classified based on their relevance to the important indicators provided by the WHO PHC Monitoring framework. | Thank you for your suggestion.  However, we preferred to classify the retrieved papers based on the themes of the WHO monitoring framework instead of specific indicators due to the high number and very specific nature of WHO indicators. Hence, we have aligned our analysis with the framework's key indicators. For example, the titles and subtitles in the results section were structured to reflect the WHO framework's guiding principles. While it was tricky to insert references in the figure, we used the WHO framework figure to categorise and present these findings related to the challenges. We used a table to extract successes. These approaches enabled us to synthesise the available evidence using the WHO framework, even though the studies were not strictly framed by it. |
| 2. | In terms of governance, there is increasing focus on organising service delivery at the district level. Accordingly, Ethiopia's Woreda or District level transformation agenda has emphasised the need to create Primary Health Care Unite (PHCU) comprising five satellite HPs, referral HCs and primary hospital with activities planned and monitored at District /Woreda level. The authors do not seem to follow this definition of PHCU. | Thank you for this critical comment.  We believe we have adequately addressed the key attributes of PHCU, including the challenges and successes of HEP, achievements and challenges faced by HEWs, the referral system, and overall efficiency, as reported in the studies and available evidence. These elements are discussed in detail within the manuscript, and we hope they provide a comprehensive overview of the relevant PHCU attributes.  In addition to this, we have included a paragraph to strengthen the discussion in the governance and leadership section and included appropriate citations. |
| 3. | In any case it is important to monitor progress and to find out the successes and challenges of implementing this extremely important initiative that would be necessary for strengthening continuum of care and the referral system. Hence, I would like to recommend inclusion of review of studies conducted in relation to district level management, such as that of AMREF: I.E (Woreda Level Deep Dive Assessment to Inform Integrated Health System Strengthening (IHSS) Investment, MERQ Consultancy PLC in Collaboration. | We appreciate your suggestion to include these important works.  We recognised this document's relevance and used evidence to strengthen our discussion. We also reference these files as suggested by the authors. |
| 4. | In terms of specific needs of the population, most of the papers reviewed described important areas of PHC services, but very few studies present findings of investigations that describe the actual situation in the country. It is noted that six studies described the importance of tailoring healthcare services and interventions to meet the specific health needs of a population. However, where is the evidence with regard to the actual situation in Ethiopia in this regard? | Thank you for the insightful suggestion.  We agree that there is a noticeable lack of studies that directly investigate the actual situation in Ethiopia.  Regarding the question on evidence specific to Ethiopia, we acknowledge that emerging studies and reports from national health programs suggest ongoing efforts to adapt PHC services to the specific health needs of different regions. These efforts include the integration of local health priorities into service delivery plans and training healthcare workers to address community-specific health challenges. However, these issues are addressed in the results section based on the recommendation of the WHO framework to reduce duplication. |
|  | **Discussion:** |  |
| 1. | The discussion lacked specific evidence to show the situation in the country due to a shortage of focused studies based on WHO's PHC monitoring framework and specifically to guide the implementation of important initiatives. | We believe we have included adequate detail on our research objectives. However, it is worth indicating that there are evidence gaps in studies based on WHO's PHC monitoring framework to guide the implementation, which we have indicated in this version. |
| 2. | - The paper has rightly emphasised the need for multi-sectoral approach to meet the dynamic population health needs. This study illustrates that multi-sectoral engagement and public-private partnerships within PHC systems remain fragmented and inconsistent. Only two papers looked into this issue, and they are not designed to evaluate the contribution of the approach. Presenting country's specific experience such as the Health Sector Development Program (HSDP) a brief review of Ethiopia's "Seqota Declaration" which could serve as exemplary strategy for muli-sectoral approach if implemented at scale. | Thanks for the suggestion.  It is valid to present the country's context regarding multi-sectoral approaches. We have included a paragraph discussing this in the revised manuscript. |
| 3. | The importance of community participation is noted. From my own past experiences working in a program that supported strengthening of the HEP whose primary focus has been on creating access to primary health care services. It improved equity and quality of health care through utilisation of essential health services, and building community ownership particularly in empowering women, as evidenced by achieving most of the health-related Millennium Development Goals (MDGs) has been great achievement of Ethiopia. But the sustainability of successes in this area needs properly structured monitoring and continued external reviews. | Thank you so much for the supporting statement.  Yes, community participation is one of the PHC pillars and well discussed in our paper |
| 4. | - The statement such as "The strength of this scoping review is that it provided a detailed landscape of PHC in Ethiopia by highlighting the successes, challenges, and pathways towards UHC and health security using the WHO's PHC Monitoring Framework" cannot be accepted. Limitations in finding relevant studies needs to be emphasised. | Thanks.  Presenting the strength of our study is equally important as presenting our limitations. We revised it and took the necessary caution to balance our study's strengths and limitations. |
| 5. | - The statement … "Results from this study provide a comprehensive and structured understanding for the researcher, policymakers and program experts to identify, research, and resolve the challenges of achieving UHC and health security". This is acceptable with the limitation of the review well explained. | Thanks |
| 6. | - Clear recommendations are expected from the authors. There is a need to highlight areas that require in-depth studies: Such as the monitoring of the implementation of Woreda/District level transformation agenda, a very important initiative to strengthen Primary Health Care Unit establishment and functioning. | Thanks for this valid suggestion. Recommendations related to the monitoring of the implementation of the Woreda/District level transformation agenda |
| 7. | - As it could be understood from browsing the references listed in this paper there is lack of periodic assessment of the PHC system particularly to determine capacity of PHC for implementing UHC in Ethiopia. One of the most relevant studies cited is reference number 81 (Eregata GT, Hailu A, Memirie ST, Norheim OF. Measuring progress towards universal health coverage: National and subnational analysis in Ethiopia. BMJ Global Health 2019). In this paper it is stated that "… Few national and subnational studies monitor UHC in low-income countries and there is none for Ethiopia". | Thanks a lot; as you know, this has been the case in our scoping review, too. |
| 8. | - Concerning service integration, a need for political commitment and investment at all health system levels for Strengthening NCD prevention and control through PHC in Ethiopia requires emphasis as part of the recommendation. | We accept your suggestion to add a recommendation point regarding Strengthening NCD prevention and control through PHC in Ethiopia. |
|  | **Conclusion:** |  |
| 1. | - It is noted that the objective of this review is to evaluate success and challenges towards achieving UHC by using WHO's PHC Monitoring Framework. However, the finding is that studies designed to monitor the progress underway to ensure the implementation of relevant and attractive health polices is almost non-existent. | The objective of this scoping review was not to entirely focus on the monitoring and evaluation studies. We believe the success and challenges of PHC, and its care provision could be assessed in other epidemiologic studies, so we prefer to leave the section as it is. |
| 2. | -There is a need to revise the presentation of this manuscript to show to policymakers and health partners the need for establishing a strong monitoring system by stressing the scarcity of data based on WHO's PHC Monitoring Framework. Objective of the review should then be to evaluate availability of published studies to review the Successes, Challenges, and Pathways Towards Universal Health Coverage and Health Security Using the WHO's PHC Monitoring. | We do appreciate the suggestion.  However, based on the WHO monitoring framework, this scoping review provides comprehensive evidence for policymakers and partner organisations to gauge progress towards UHC. |
| 3. | - Ethiopia's experience in regularly reviewing HSDP by using external review till the end should have been maintained. It should be remembered that it has been the review of HSDP I that initiated the Health Extension Program (HEP) and subsequent reviews helped in strengthening its implementation. | Thank you for the suggestion.  However, adding this recommendation is beyond the scope of this manuscript, and we respectfully defer on this point. |
|  | **Reviewer 2-** |  |
| 1. | General comment: |  |
|  | The synthesis of the literature offers a valuable foundation for understanding the landscape of primary health care in Ethiopia. However, I would like to raise a few points regarding the scope and depth of the analysis presented in your review. While the detailed summaries of the studies offer essential insights, it appears that the review primarily focuses on these narratives without incorporating a broader range of data elements like primary health care in hospital settings. This limitation could potentially hinder the overall depth of insight and analysis that can be derived from your findings. The inclusion of more extensive data would not only enrich the discussion but also foster a more nuanced understanding of the trends and patterns observed in the literature. | Thanks for this constructive comment.  In the PHC delivery model section, we have included information regarding task-sharing between primary care settings and hospitals, which is designed to increase service access by reducing transportation stress and costs. However, it is worth noting that the Ethiopian PHC system is primarily designed to provide PHC at the PHCU, including health posts and health centres. |
| 1. | The time frame covered in your review—from 1998 to the present—provides a significant opportunity for reflection on the advancements and developments that have taken place within the primary healthcare system in Ethiopia over the past three decades. This longitudinal perspective is crucial, as it allows for the identification of both improvements and persistent challenges in the healthcare landscape. | Thanks for the constructive comment |
| 2. | To further enhance the impact and utility of your findings, I encourage you to incorporate additional data elements that could address the gaps and limitations identified in the reviewed studies. For instance, exploring the strengths of the primary health care system in Ethiopia, along with an examination of primary health care service delivery in hospital settings, could provide a more balanced view of the current state of health care delivery. By integrating these elements into your review, you would not only strengthen your analysis but also offer readers a more comprehensive understanding of the current landscape in your area of study. This would ultimately contribute to the ongoing dialogue surrounding primary health care in Ethiopia and help inform future efforts aimed at improving health outcomes. | Thank you for the suggestion. We agree that presenting the broader context of Ethiopia's PHC strengths is important. So, we have used the Health Sector Development Program (HSDP) and other important evidence to show the broader PHC service delivery in our discussions. |
|  | Title: |  |
| 1. | - The title looks slightly overwhelming due to its length and complexity. The multiple concepts it introduces primary health care (PHC), successes, challenges, pathways, universal health coverage (UHC), health security, and the WHO framework make it dense. Simplifying the title or breaking it into more focused elements could enhance readability and clarity, helping readers quickly grasp the core focus of the manuscript. Additionally, the inclusion of both UHC and health security may be seen as broad, potentially requiring more specifics.  - It would be beneficial to streamline the content by focusing on the central themes and eliminating redundancy. Breaking the title into more focused elements will help readers immediately grasp the main subject without being overwhelmed. Additionally, narrowing the scope to either "Universal Health Coverage" or "Health Security" would allow for a more precise focus, given that both are broad and may not be fully addressed in a single review. A more concise version could emphasise the key concepts of the review (Ethiopia's PHC) and its alignment with global health goals.  - Therefore, I suggest the title to be: "Strengthening Primary Health Care in Ethiopia: A Scoping Review of Successes, Challenges, and Pathways Towards Universal Health Coverage using WHO's Monitoring Framework". | Suggestion accepted.  The title has been modified to read as:  Strengthening Primary Health Care in Ethiopia: A Scoping Review of Successes, Challenges, and Pathways Towards Universal Health Coverage Using the WHO's Monitoring Framework |
|  | Abstract: |  |
| 2. | The abstract would benefit from more concrete suggestions for overcoming the identified challenges, such as strategies for improving resource allocation, addressing rural inequities, and fostering stronger governance and service integration.  - In addition, offering policy recommendations based on the findings could provide practical guidance for decision-makers in Ethiopia to effectively address the challenges and build on the successes of the PHC system. | Thanks for this thoughtful suggestion.  We revised the abstract section to reflect concrete conclusions and suggestions for policymakers. |
|  | Introduction: |  |
| 1. | To enhance the introduction, it would be beneficial to incorporate more specific details about the ongoing challenges Ethiopia faces in its PHC system, especially in terms of regional disparities, resource allocation, and workforce distribution being impacted by narrow fiscal space. These aspects would provide a more balanced view of both the successes and challenges within the system. Additionally, a more in-depth discussion of the health extension program and its impact on improving health access to basic health services and outcomes in underserved areas would strengthen the narrative and provide concrete examples of Ethiopia's PHC approach in practice. | We appreciate the thoughtful suggestion.  We believe the broader PHC and UHC contexts may require a more profound discussion to illustrate the progress and challenges better. However, we chose to limit the introduction and address the specific details about Ethiopia's ongoing challenges in the result and discussion sections.  Nevertheless, addressing regional disparities, resource allocation, and workforce distribution being impacted by narrow fiscal space is important. We have revised the introduction to address these contexts and provide a more balanced view.  Our results section, discussion, and many references cited in this scoping review address more details about HEP. We intentionally limited the details of HEP. |
| 2. | Including case studies or specific instances of multi-sectoral collaboration, both successful and challenging, would offer readers a more understanding of how these partnerships work in practice and their impacts. | Comment accepted.  It is valid to present the country's context regarding multi-sectoral approaches, and we have used the HSDP and "Seqota Declaration" to show the multi-sectoral approaches of the countries' PHC. |
|  | Methods: |  |
| 1. | - To improve the clarity and robustness of the methodology, expanding the inclusion criteria to include grey literature, such as government reports, policy documents, and publications from development partners or non-governmental organisations, could enrich the review by providing context-specific insights that are often omitted from peer-reviewed studies. | Thank you for your valuable suggestion.  We believe the methodology section provides sufficient detail regarding the methodology used in this review and is robust.  Regarding the inclusion of grey literature, such as government reports and policy documents, although we believe they enrich the review, we believe it is not feasible to include them at this stage, so we prefer to maintain the current approach for now. However, we used these documents to enrich our discussion. |
| 2. | - To minimise potential language bias, it might be worth considering the inclusion of studies published in other languages, such as Amharic, or providing a justification for excluding non-English studies if this is not feasible.  Additionally, involving more authors in the data extraction and verification process would enhance the reliability of the results and reduce the potential for bias or error. Finally, providing a clearer description of the thematic analysis process, including how themes were identified and the specific subthemes used for categorisation, would help. | Thank you for your feedback.  We believe that most peer-reviewed publications from Ethiopia are published in English. While we understand that some authors may publish findings in Amharic for research translation purposes, including Amharic evidence could lead to duplicates, as these will likely be excluded during the review process.  Regarding data quality assurance and reliability, while we understand that quality appraisal criteria are optional in conducting a systematic review, we took several steps to ensure the accuracy and integrity of the extracted data. This included thorough checks and re-checks of the data with our co-authors and the senior author, aiming to minimise bias and errors throughout the process.  As for the thematic analysis and themes, we have clearly indicated that the key focus of the WHO PHC monitoring framework and the PHC strategic and operational levers formed the foundation for the themes used to narrate our findings. We believe this approach provides a clear structure for presenting our results. |
|  | **Results:** |  |
| 1. | Addressing Placeholder References: The manuscript should ensure that all references (e.g., Error! Reference source not found") are citations to maintain the quality and professionalism of the manuscript. | Thank you for pointing this out.  We apologise for the oversight and understand the importance of maintaining the quality and professionalism of the manuscript. We will address the placeholder references (e.g., "Error! Reference source not found") and ensure all citations are correctly formatted.  We appreciate your attention to this detail. |
| 2. | Clarification and Elaboration on Key Findings: It would enhance the clarity and impact of the results to provide more detailed, concrete examples, especially in the discussion of challenges like the low integration of NCD and palliative care services. Specific case studies or quantitative data would strengthen these claims and offer readers a clearer understanding of the situation. | We accept your suggestion.  We have added further clarity to the discussion of challenges, such as the low integration of NCD and palliative care services, by including relevant quantitative data to support our points. |
| 3. | Expansion on Digital Health: Given the increasing importance of digital technologies in healthcare, the section on digital health should be expanded to provide a more in-depth analysis. Discussing successful mHealth interventions, their impact on health outcomes, and the barriers to digital health adoption would make this section more robust. Addressing the role of digital health in improving service delivery during the COVID-19 pandemic is particularly relevant and could be expanded. | Thank you for your valuable comment.  Given its increasing importance in healthcare, we believe we have already included the significant barriers to implementing digital health. For example, in the "Digital Technology for Health" section, we discussed key barriers such as users' digital literacy, critical digital infrastructure challenges, competing projects, limited internet connectivity, and the low attitude of health professionals toward digital health, with proper citations.  Additionally, in the "Health Information" section, we have addressed the importance and barriers of digital health and information systems.  We agree that revising and expanding the section would strengthen the discussion. We will revise this part, accordingly, as reflected in our tracked revisions. |
| 4. | Structuring Health Workforce Findings: The section on the health workforce could be more structured by categorising the challenges into distinct themes such as staff shortages, turnover, burnout, and training gaps, as well as successes in health workforce development and deployment. This would improve readability and allow for a more systematic discussion of the issue | Thank you for your suggestion.  While we understand the importance of structuring the health workforce findings into distinct themes, we believe the current structure effectively highlights the interconnected characteristics of professional development training gaps, staff burnout, turnover, and subsequent staff shortages. Categorising them separately might risk oversimplifying the complex and overlapping issues within the health workforce. |
| 5. | Strengthen Analysis on Health Security: There is a noted gap in the analysis of health security in the PHC system, which could be addressed more explicitly. Expanding on the relationship between health security and PHC, including potential risks and the integration of health security measures within the PHC framework, would be valuable for readers concerned with health system resilience. | Thank you for this critical comment.  Our study highlights that fragmented health systems and limited resources hinder health security and PHC synergy. We faced some challenges in providing detailed attributes of health security within the context of Ethiopian PHC due to a significant evidence gap in health security as a subject of research and implementation measures. Ethiopia's PHC system also lacks comprehensive data on health security threats and risk behaviours, which we believe could be an important area for future research. |
| 6. | Provide Clearer Data on Service Coverage and Inequality: The results section briefly touches on service coverage and inequality but lacks a more detailed breakdown. Adding figures or examples to illustrate the extent of disparities in access to PHC services across different regions, urban vs. rural areas, and among different population groups would further underscore the gaps and provide more actionable insights. | Thank you for your suggestion.  We could add more details and data regarding service coverage, but we had to limit our findings to what has been addressed in the included studies. The studies we reviewed primarily provided data on specific aspects of service coverage, such as immunisation, RMNCH services, and community-based health insurance, in our results section discussions. While it is understood that it is impossible to address all coverage-related data and inequality in a single paper, we hope the current level of detail and included data provide a sensible understanding of service coverage and regional inequities in PHC's major service areas.  In addition, we highlight the broader challenges of improving service coverage in Ethiopia's PHC towards UHC, such as resource limitations, health system fragmentation, donor-driven vertical programs, and diverse health financing mechanisms. |
| 7. | Improving Transitions between Sections: The review could benefit from smoother transitions between themes, ensuring that the findings related to each topic are logically connected and flow coherently from one section to the next. This would improve readability and help the reader easily follow the narrative | Comment accepted, and revisions made |
|  | Discussion: |  |
| 1. | To strengthen this discussion section, it is recommended to include more detailed examples of the political and governance challenges at various levels of the healthcare system, particularly focusing on how regional and district-level leadership can be more effectively engaged in the implementation of PHC policies. | Comment accepted.  We added a paragraph in the discussion section, as suggested. |
| 2. | Additionally, expanding the analysis of the barriers to service integration, particularly related to infrastructure and human resources, would provide a more holistic understanding of the challenges Ethiopia faces in expanding and sustaining PHC services. Given the importance of digital health and ICT in the future of PHC, a more detailed exploration of Ethiopia's ICT challenges – such as internet access, data quality, and technology adoption would be useful. | Thank you for the suggestion. We believe that service integration crosses a wide range of services, and we have already provided a balanced level of detail on the topic. However, we have included some more details in the discussion section regarding the challenges of ICT services and the sustainability of PHC interventions. |
| 3. | Finally, the manuscript could benefit from a more explicit connection between the identified challenges and potential solutions, such as recommending specific actions or policy adjustments that could address the governance issues, improve infrastructure, and enhance multi-sectoral coordination. Expanding the call for empirical research to include underexplored topics like the impact of service integration on PHC system resilience in emergencies and detailing how to engage the broader community in sustaining PHC, would help make the paper even more actionable for future research and policy development. | Thanks.  We tried our best to make a good flow of the paragraphs and sections. |
|  | Conclusion: |  |
| 1. | To enhance the conclusion section, it would be helpful to provide more specific, actionable recommendations for addressing the highlighted challenges. For instance, the manuscript could offer examples of successful governance reforms or multi-sectoral collaborations from other countries that could be adapted to the Ethiopian context. Concrete strategies for improving financial governance, such as detailed steps to enhance funding allocation or innovative funding models for rural health services, could be included. The mention of NCDs, palliative care, and infrastructure gaps should be expanded upon with more concrete policy suggestions, such as prioritising these areas in health planning and resource allocation. | Thanks for the suggestion.  The conclusion section has been revised to reflect some of the suggestions. |
| 2. | Additionally, the role of evidence-based decision-making in overcoming these challenges could be incorporated, perhaps suggesting the need for strengthened data collection and monitoring systems to guide policy adjustments. | Thanks for the suggestion.  We revised the conclusion section and added the suggested points |
| 3. | Lastly, while the empowerment of communities and local leaders is an important point, the manuscript could be improved by offering more details on how these efforts can be scaled, sustained and integrated into the broader PHC system, along with examples of effective community engagement models. By adding these specifics, the conclusion would offer a more roadmap for future progress in Ethiopia's PHC system. | Thanks for the suggestion.  We revised the conclusion section and added the suggested points |
|  | References |  |
| 1. | • **Duplication of Citations**: There is a notable duplication of references, particularly with the *Assefa et al.* (2020) study being cited multiple times (References 5, 11, and 13). This redundancy could confuse readers, making it unclear whether these references refer to the same or different studies. Each reference should be cited only once, and if multiple works from the same author are cited, they should be clearly differentiated using proper citation styles. | Thanks for this. Duplicates removed |
| 2. | **Missing Citations**: Some references listed in the reference section do not appear to be cited in the text. For example, the reference by *Lavers (2021)* (Reference 2) is not mentioned within the in-text citations, even though it is a key source regarding universal health coverage in Ethiopia. Similarly, studies like *Tadesse et al. (2021)* (Reference 14) and *Verguet et al. (2021)* (Reference 19) may also lack corresponding in-text citations. The author should ensure that every reference listed is properly cited in the text and that all cited studies are included in the reference section.  • **Inconsistent Formatting**: There are inconsistencies in the formatting of references. For example, *References 1 and 36* list the publisher or organisation in a somewhat inconsistent manner (e.g., "World Health Organization" versus "WHO" without clarification). A standardised format for listing publishers or organisations should be adopted throughout the reference section to ensure consistency. | Thank you for your feedback!  We're not sure how it was overlooked, but we can confirm that these references were presented as corresponding in-text citations:   - Lavers (2021) is cited on page 4, paragraph 1, line 6. - Tadesse et al. (2021) is cited on page 4, paragraph 2, last line. - Verguet et al. (2021) is cited on page 7, paragraph 2, last line.   We appreciate the suggestion regarding inconsistencies in the references and have addressed them throughout the manuscript. |
| 3. | **Lack of Volume and Issue Numbers**: Several references are missing volume or issue numbers, such as *Reference 86 for Aregay et al. (2023)*. This inconsistency may detract from the professionalism of the reference list and could make it more difficult to locate the sources.  • **Inconsistent Capitalisation and Journal Name Formatting**: The referencing style lacks consistency in terms of capitalisation and the presentation of journal names. For example, some journals have their names fully capitalised, such as *HEALTH EXPECTATIONS* (Reference 88). This formatting issue should be corrected to adhere to a standardised style, ensuring that journal names are presented consistently. | Thank you for your feedback.  We have reviewed the references and ensured that all missing volume and issue numbers are correct.  We have addressed the references' inconsistencies and ensured all citations are streamlined. |
| 4. | • **Use of Preprints**: Some references listed in the manuscript are preprints (e.g., medRxiv for References 100 and 101). While preprints are useful for sharing emerging research, they are not peer-reviewed articles. | Thanks for raising this issue.  We were able to find the peer-reviewed version and have now excluded the preprints |
